# Sodium β-Diketonate Glyme Adducts as Precursors for Fluoride Phases: Synthesis, Characterization and Functional Validation

**DOI:** 10.3390/molecules27196282

**Published:** 2022-09-23

**Authors:** Nishant Peddagopu, Anna L. Pellegrino, Carmela Bonaccorso, Patrizia Rossi, Paola Paoli, Graziella Malandrino

**Affiliations:** 1 Dipartimento di Scienze Chimiche, Università di Catania and INSTM UdR Catania, V.le Andrea Doria 6, 95125 Catania, Italy; 2 Dipartimento di Scienze Chimiche, Università di Catania, V.le Andrea Doria 6, 95125 Catania, Italy; 3 Dipartimento di Ingegneria Industriale, Università di Firenze, Via Santa Marta 3, 50136 Firenze, Italy

**Keywords:** sodium complexes, second-generation precursors, single-crystal structure, sodium fluoride, MOCVD, sol-gel method

## Abstract

Very few sodium complexes are available as precursors for the syntheses of sodium-based nanostructured materials. Herein, the diglyme, triglyme, and tetraglyme (CH_3_O(CH_2_CH_2_O)_n_CH_3_, *n* = 2–4) adducts of sodium hexafluoroacetylacetonate were synthesized in a single-step reaction and characterized by IR spectroscopy, ^1^H, and ^13^C NMR. Single-crystal X-ray diffraction studies provide evidence of the formation of the ionic oligomeric structure [Na_4_(hfa)_6_]^2−^•2[Na(diglyme_2_]^+^ when the diglyme is coordinated, while a mononuclear seven-coordinated complex Na(hfa)•tetraglyme is formed with the tetraglyme. Reaction with the monoglyme (CH_3_OCH_2_CH_2_OCH_3_) does not occur, and the unadducted polymeric structure [Na(hfa)]_n_ forms, while the triglyme gives rise to a liquid adduct, Na(hfa)•triglyme•H_2_O. Thermal analysis data reveal great potentialities for their applications as precursors in metalorganic chemical vapor deposition (MOCVD) and sol-gel processes. As a proof-of-concept, the Na(hfa)•tetraglyme adduct was successfully applied to both the low-pressure MOCVD and the sol-gel/spin-coating synthesis of NaF films.

## 1. Introduction

Sodium-based functional materials are of great interest due to their high technological potentials, e.g., in thermoelectrics (e.g., Na_x_CoO_2_) [1,2], as superconductors (e.g., Na_x_CoO_2_·yH_2_O) [3], in dielectrics (e.g., NaNbO_3_) [4], and in piezoelectrics (K_1−x_Na_x_NbO_3_) [5,6]. Lately, sodium has also attracted great attention for its use in sodium-ion batteries, a less expensive alternative to lithium-ion batteries [7,8]. 

In addition to oxides, sodium metal fluorides in the form of thin films show peculiar characteristics that make them promising systems for different optical applications, including light generation and wave guide devices [9]. Recently, the metal fluoride mixed NaREF_4_ (RE = Y or Gd) systems have attracted renewed attention for their application in photovoltaic cells, due to their potential use as a host matrix for luminescent lanthanide ions, especially for emissions visible upon near-infrared (IR) excitation due to the phenomenon of energy up-conversion [10,11,12,13].

For the above-mentioned technological applications, there is a demand for materials in form of nanostructured thin films. Sodium fluoride has been deposited as thin films using pulsed laser deposition [14], RF-sputtering [15], metalorganic chemical vapor deposition (MOCVD) [16,17,18] and, more recently, atomic layer deposition (ALD) [19]. 

Concerning the MOCVD approach, β-diketonate complexes are common precursors, while metal alkoxides or acetates are more frequently used in solution-based methods [20,21,22,23]. Overall, for application in conventional MOCVD, precursors must be volatile, have the necessary stability to reach the deposition site, and cleanly decompose to produce the desired material. An added advantage of the metal β-diketonate complexes is that they often demonstrate higher hydrolytic stability than their alkoxide counterparts. A variety of β-diketonate complexes of both main groups and transition metals have been used to deposit films of metals [24], oxides [25], sulphides [26], and fluorides [27]. As ligands, β-diketonates show a wide range of coordination modes, the degree of bridging being determined by the metal center, the steric encumbrance of the ligand, and the presence of Lewis bases. The oligomerization degree of some large ionic radius metals, e.g., groups 1, 2, and the lanthanides, can be broken by coordinating one Lewis base, e.g., the glyme ligands [23,28]. Recent studies have also shown that sodium-•glyme based electrolytes are promising candidates in the development of sodium ion batteries due to their compatibility with conventional graphite electrodes [29,30,31,32]. We have previously detected and tested the promising properties of a large family of single-source precursors of the type M(hfa)_n_•glyme for fabricating high-quality alkaline-earth [33,34,35], and rare-earth [36,37,38] fluoride films. These precursor architectures consist of a fluorinated β-diketonate metal moiety, where the metal coordinative deficiency is saturated by various Lewis base ligands, such as glyme, producing most of the monomeric, water-free, stable, volatile, and low melting point precursors [23,28,33,34,35,36,37,38]. All these properties are of relevance, and, in particular, low melting point precursors represent the most desirable sources due to the more significant and stable vapor pressure, since their liquid nature under process conditions prevents any effects of the crystallite size on the evaporation and, hence, on the film growth rate.

In the literature, there are some reports on the formation of sodium complexes [39,40], but, to our knowledge, only the following have been proposed as MOCVD precursors: the Na(tmhd)•phen and Na(tmhd)•bipy (Htmhd = 2,2,6,6-tetramethyl-3,5-heptanione, phen= 1,10-phenanthroline, bipy = 2,2′-bipyridyl) complexes [41]. Nevertheless, these complexes do not contain fluorine in their architecture, and furthermore their mass transport studies show thermal instability.

The main issues in the synthesis of stable sodium precursors are steric and electronic factors. In fact, the alkali metals have a large ionic radius and thus a large coordination number, which involves, generally, an increase in the metal–ligand distances of the complex, resulting in a decrease in the force constant and in the stability of the complex itself. 

In this context, we focused our attention on the synthesis of new sodium single-source precursors suitable for the growth of NaF thin films.

Here, we report the synthesis of novel sodium complexes of the type “Na(hfa)•glyme”, (Hhfa = 1,1,1,5,5,5-hexafluoro-2,4-pentanedione, glyme = monoglyme (1,2-dimethoxyethane), diglyme (bis(2-methoxyethyl)ether), triglyme (2,5,8,11-tetraoxadodecane), and tetraglyme (2,5,8,11,14-pentaoxapentadecane)), with the glymes performing like crown ethers in terms of coordinating/solvating ions, i.e., in terms of chelating properties. Single-crystal X-ray diffraction experiments provided evidence of interesting coordination moieties. In particular, the Na(hfa)•tetraglyme adduct single-crystal structure was previously communicated [42]. They were also characterized by FT- IR spectroscopy, ^1^H, and ^13^C NMR. The functional validation of these complexes as precursors of Na-F films was addressed by applying the Na(hfa)•tetraglyme adduct to the MOCVD and sol-gel processes for sodium fluoride film formation.

## 2. Results and Discussion

Novel sodium complexes were synthesized, in a single-step reaction, from the sodium hydroxide, hexafluoroacetylacetone, and glyme ligands in dichloromethane. The mixture was refluxed under stirring for 1 h through the following equations (Equations (1) and (2)):


NaOH + Hhfa + L → Na(hfa)•L + H_2_O (1)

NaOH + Hhfa → Na(hfa) + H_2_O (2)

**1**  [Na(hfa)]_n_
**1a**  “Na(hfa)•monoglyme”
**2**  [Na_4_(hfa)_6_]^2−^•2[Na(diglyme)_2_]^+^
**3**  Na(hfa)•triglyme•H_2_O
**4**  Na(hfa)•tetraglyme

After the solvent evaporation, the precursors **2** and **4** were obtained as white crystals and melted respectively at 107 ± 1 °C and 64 ± 1 °C. Compound **1a** had a white, sticky, crystal-like consistency, while precursor **3** was a colorless oil at room temperature. All the adducts were mostly soluble in common organic solvents such as ethanol, dichloromethane, and acetone. Complexes **1**, **2**, and **4** are non-hygroscopic and can be handled in air. Thus, our synthetic strategy produced anhydrous adducts, except for adduct **3**, with high yields (80–90%) in a single and low-cost route from commercially available chemicals. 

An in-depth analysis of adduct **1a** proved that, under these synthetic conditions, the monoglyme molecule is actually not coordinated. 

### 2.1. Single-Crystal Structures 

**Figure 1:**
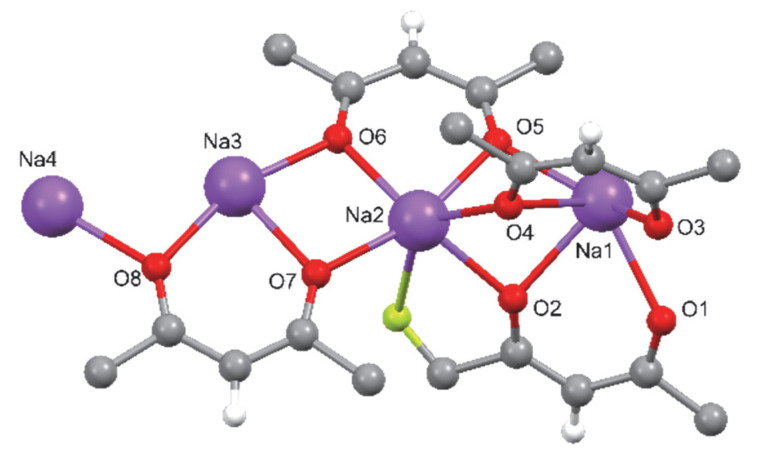
Ball and stick view of the asymmetric unit of **1**. Some fluorine atoms have been omitted for the sake of clarity.

**Figure 2:**
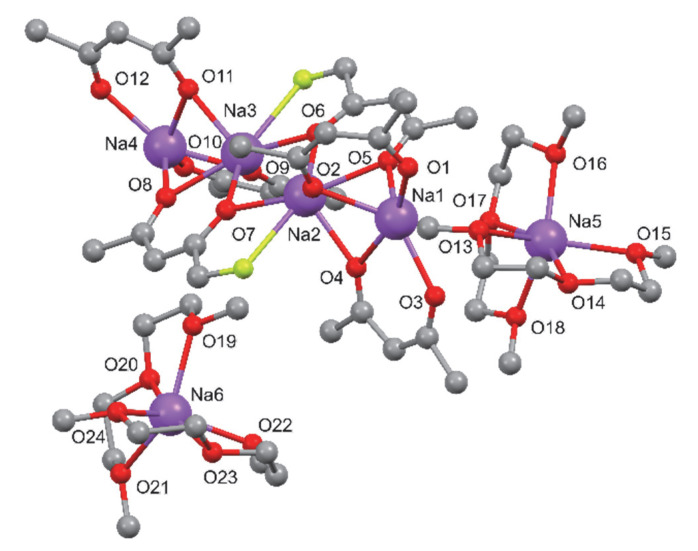
Ball and stick view of the asymmetric unit of **2**. For the sake of clarity, hydrogen atoms and some fluorine atoms have been omitted.

**Figure 3:**
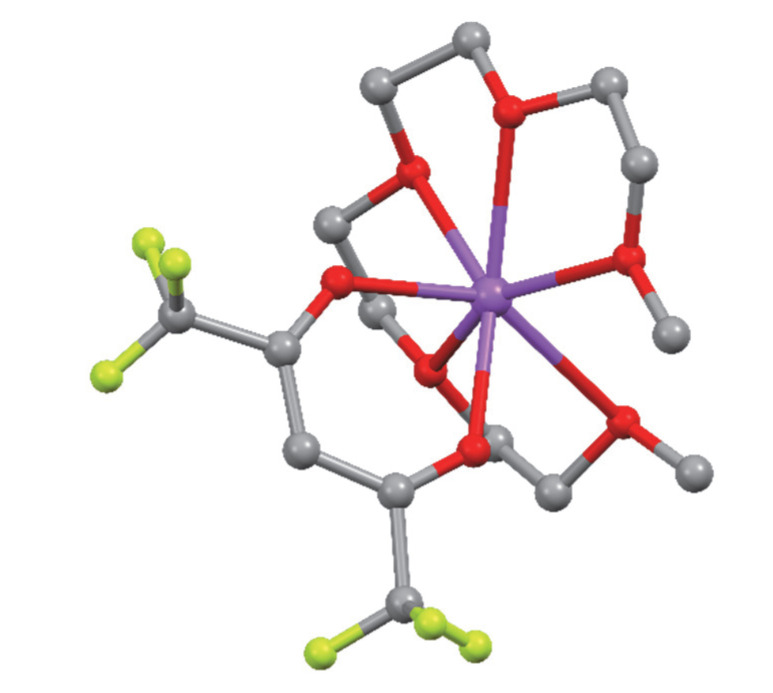
Ball and stick view of the asymmetric unit of **4** CATTAV. For the sake of clarity, hydrogen atoms have been omitted.

The structures of compounds **1** and **2** were determined by single-crystal X-ray diffraction analysis. In addition, their coordination schemes were compared with the already known structure of the Na(hfa)•tetraglyme complex **4** (Cambridge Structural Database refcode = **CATTAV**) [42]. In Figure 1, Figure 2 and Figure 3 the ball and stick views of the asymmetric unit of the compounds **1**, **2,** and **4 CATTAV** are reported.

In the asymmetric unit of **1,** four sodium cations and four hfa anions are present. Five oxygen atoms coordinate all the sodium cations, and their coordination spheres are completed by several fluorine atoms ranging from 2 to 4, giving rise to a coordination number in the 7–9 range (in particular, more Na2 and Na3 are hepta-coordinated, Na4 is octa-coordinated, while Na1 is nona-coordinated). The Na-X distances are reported in Supplementary Materials: Table S1.

**Figure 4:**
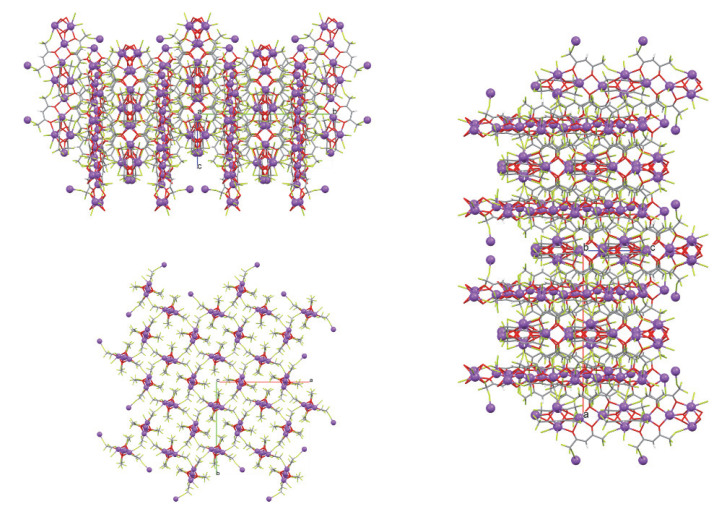
Views along the a, b, and c axes of crystal packing of **1** (**top-left**, **right**, and **bottom-left**, respectively).

The oxygen atoms of two hfa anions (O1, O2, and O3, O4) act as μ3-bridging atoms toward three sodium cations, while the oxygen atoms of the other two hfa anions (O5, O6, and O7, O8) bridge two metal cations (Figure S1). Due to these bridging atoms’ presence, a 3D polymer is formed (Figure 4).

**Figure 5:**
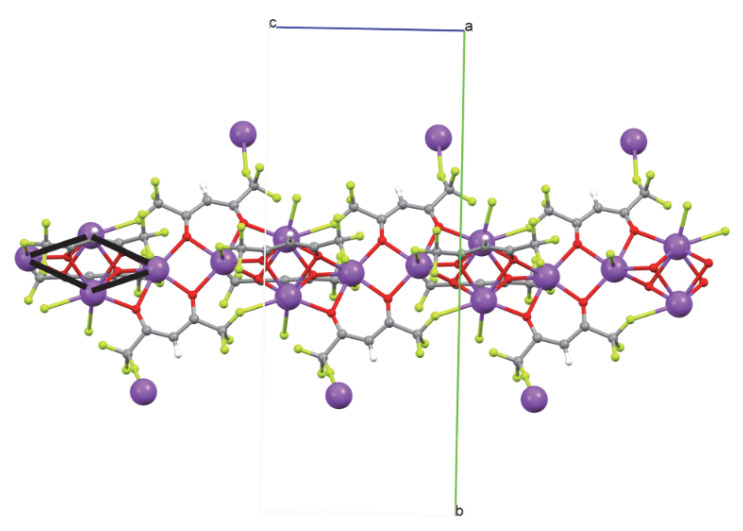
The rhombus defined by four proximal sodium atoms in the structure of **1** is shown in black.

Inside the 3D polymer, it is possible to localize a group of four sodium cations which occupies the rhombus’ vertices (see Figure 5 and Table S1). These rhombuses form a chain that propagates along the c axis (Figure 5).

**Figure 6:**
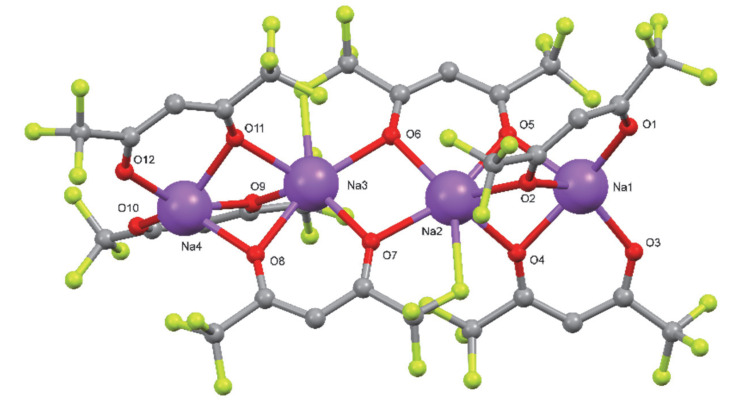
Ball and stick view of the Na_4_(hfa)_6_^2−^ anion in **2**.

In the asymmetric unit of compound **2**, two Na(diglyme)_2_^+^ cations and one Na_4_(hfa)_6_^2−^ anion are present. In the Na(diglyme)_2_^+^ cation, the sodium atom is hexa-coordinated by all the three oxygen atoms of the two diglyme molecules. Concerning the Na_4_(hfa)_6_^2−^ anion (Figure 6), all the sodium atoms are coordinated by five oxygen atoms, and, in addition to these, the central metal cations Na2 and Na3 are bonded to a fluorine atom (see Table S2). 

All the oxygen atoms of the four hfa anions of Na_4_(hfa)_6_^2−^ act as μ^2^-bridging atoms with the exceptions of O1, O3, O10, and O12, which belong to hfa anions situated at both ends of the Na_4_(hfa)_6_^2−^ unit (Figure 6). In the Na_4_(hfa)_6_^2−^ anion, the four sodium atoms are aligned. Concerning their relative distances, the couples Na1/Na2 and Na3/Na4, which are bridged by three oxygen atoms, are significantly nearer than the central one Na2/Na3 (see Table S1).

A comparison with the similar complex **CATTAV** evidenced how, starting with similar ligands (hfa in **1**, hfa and diglyme in **2**, and hfa and tetraglyme in **CATTAV**), different coordination geometries may be obtained, even if the synthetic procedure is the same. In fact, while the tetraglyme molecule contains a number of donor atoms able to fulfil the coordination needs of the sodium cation bonded to the hfa anion, this is not the case for the diglyme molecule (nor for compound **1**, where only the hfa anion is present as ligands), and completely different coordination schemes are obtained. 

Concluding, the present syntheses of adducts **2**, **3**, and **4** give us a good understanding of the polyether chain’s effect on the central metal β-diketonate structure’s coordination chemistry. Analogous coordination behavior has recently been observed for the Li and K ions using the hfa anionic ligand and the same glyme Lewis base ligands [43,44].

### 2.2. NMR Characterization

The ^1^H-NMR and ^13^C-NMR spectra of the “Na(hfa)·glyme” adducts were recorded dissolving the more polar adducts **1** and **2** in CD_3_CN, while **3** and **4** were dissolved in CDCl_3_. In any case, to make uniform the characterization of the complexes, the ^1^H-NMR and ^13^C-NMR spectra of **3** and **4** in CD_3_CN were also recorded. 

The ^1^H and ^13^C signal attribution for complexes **1**–**4**, acquired in CD_3_CN, is reported in Table 1. The complex **1** shows only a singlet due to the proton of the hfa anion (Figure S2). The ^1^H-NMR spectra of adducts **2**, **3,** and **4** (Figures S4, S6, S7, S10, and S11) show also the signals of the polyether moieties: a singlet at δ ≈ 3.3–3.4 ppm consistent with the protons of the terminal methyl groups, and the singlets/multiplets at slightly lower fields (δ ≈ 3.5–3.7 ppm) for the inner methylenic protons. For all the adducts, the singlet for the proton of the hfa anion resonates at δ ≈ 5.6–5.8 ppm. The presence of these signals at lower fields is a clear indication of the deprotonation of the hfa ligand. Moreover, both the absence of polyether signals for adduct **1** and the integration values observed for **2** and **4** (reported in Table 1) confirm the stoichiometries defined by the X-ray diffraction analysis. The ^13^C NMR spectra of all the compounds **1**–**4** (Figures S3, S5, S8, S9, and S13) were assigned using comparative arguments with data from related alkaline-earth complexes. The spectra show singlets (δ ≈ 85) for the *C*H group and the coupling of ^13^C with ^19^F nuclei: the *C*F_3_ and *C*O groups resonate at ≈ 119 ppm and ≈ 175 ppm as quartets, with coupling constant values of ^1^J_CF_ ≈ 290 Hz and ^2^J_CF_ ≈ 30 Hz, respectively. No evidence of any tautomeric equilibrium emerged from either ^1^H or ^13^C spectra for the hfa-derived moieties, the enolate (hexafluoroacetylacetonate) form being the only species observed in solution for the complexes **1**–**4**. Coordinated glymes in the compounds **2**, **3**, and **4** give signals at δ ≈ 58–59 ppm (s, O*C*H_3_, a) and singlets in the range 70–72 ppm due to the b-e O*C*H_2_, depending on the glyme length. Specific values are reported in Table 1.

### 2.3. FT-IR Characterization

**Figure 7:**
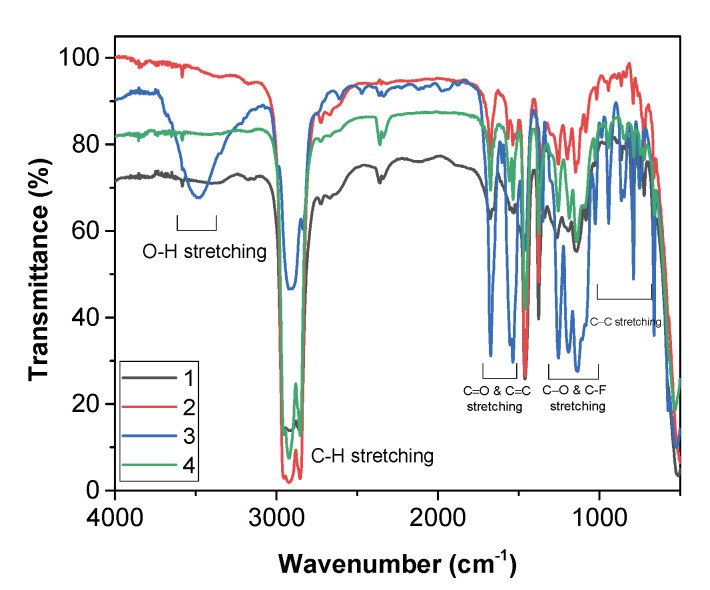
Infrared spectra of the adducts **1**, **2**, **3**, and **4** in the 4000–500 cm^−1^ range.

All of the sodium precursors were characterized by Fourier transform (FT)-IR spectroscopy in the 4000–500 cm^−1^ range. The spectra of **1**, **2**, and **4** were recorded as nujol mulls, while the spectrum of **3** was recorded on the neat sample. The FT-IR transmittance spectra (Figure 7) of the “Na(hfa)•glyme” (**2** and **4**) and of Na(hfa) (**1**) show no band at 3600 cm^−1^, indicating that no water molecules are coordinated to the metal ion, thus pointing to the metal center’s saturation, while adduct **3** shows a broad band around 3500 cm^−1^, due to H_2_O coordination. 

**Figure 8:**
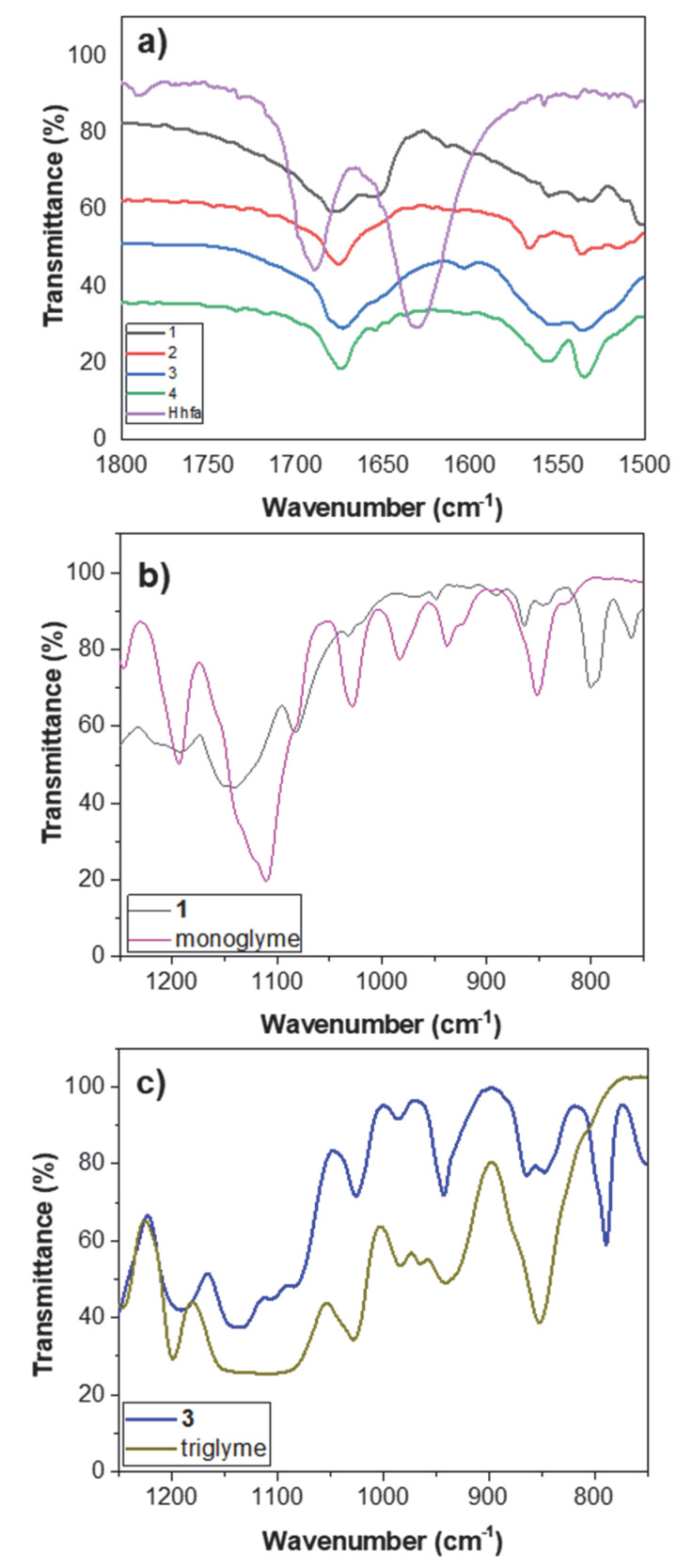
FT-IR spectra of: (**a**) the adducts **1**, **2**, **3**, and **4**, and the H-hfa ligand in the carbonyl range; (**b**) the adduct **1** and the monoglyme ligand; and (**c**) the adduct **3** and the triglyme ligand in the 1300–700 cm^−1^ range.

An enlargement of the different FT-IR spectra in the carbonyl range of the compounds **1**, **2**, **3**, and **4**, and the free H-hfa ligand is shown in Figure 8a. It is possible to observe a peak at 1689 cm^−1^, due to the C=O stretching, and one due to the C=C stretching at 1629 cm^−1^ for the enol form of the H-hfa free ligand. The peak due to the C=O stretching is slightly shifted to 1675 cm^−1^ for adducts **2**, **3**, and **4**, while for adduct **1**, a splitting of this peak can be observed, producing signals at 1678 and 1651 cm^−1^. 

The C=C stretching of all four adducts **1**–**4** is split into two peaks at 1530 cm^−1^ and 1560 cm^−1^. The broad band observed in the 1300–1000 cm^−1^ range may be related to C-O bending and/or stretching vibrations, due to coordination of the polyether, overlapped with the C-F stretching. Additionally, bands at 1015 cm^−1^, 861 cm^−1^, and 837 cm^−1^ may be associated with the glyme modes of adducts **2**, **3**, and **4**.

The C-H glyme stretching modes, lying in the 2800–3000 cm^−1^ range, overlap with nujol features, except in the case of compound **3**, whose spectrum has been recorded as a neat sample. The nujol shows peaks at 2923 cm^–1^, at 1461 cm^–1^, and 1377 cm^–1^. 

In the case of compound **1** (Figure 8b), no signals are present in the 1100–800 cm^−1^ region, as expected, considering that no monoglyme is present in this compound. For the liquid adduct **3,** the presence of the peaks around 1015 cm^−1^, 861 cm^−1^, and 837 cm^−1^ confirms the coordination of the triglyme ligand (Figure 8c).

### 2.4. Thermal Analysis

**Figure 9:**
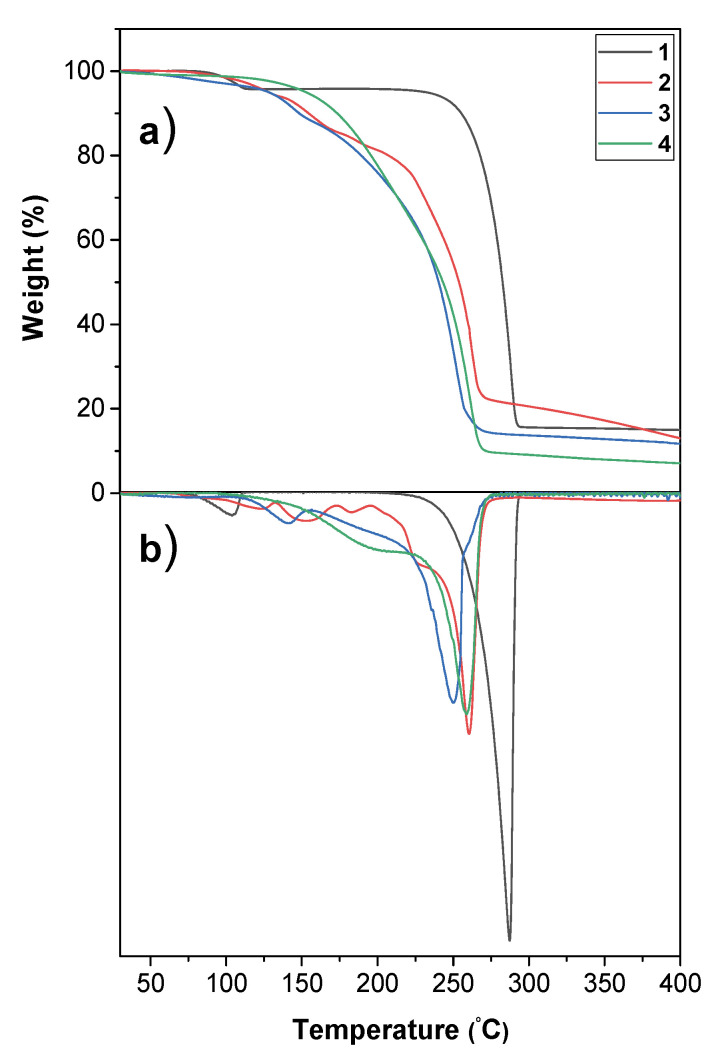
(**a**) TG, and (**b**) DTG profiles of [Na(hfa)]_n_ (**1**) and “Na(hfa)•glyme” (**2**, **3**, and **4**) under N_2_ flow at atmospheric pressure in the temperature range 30–400 °C.

**Figure 10:**
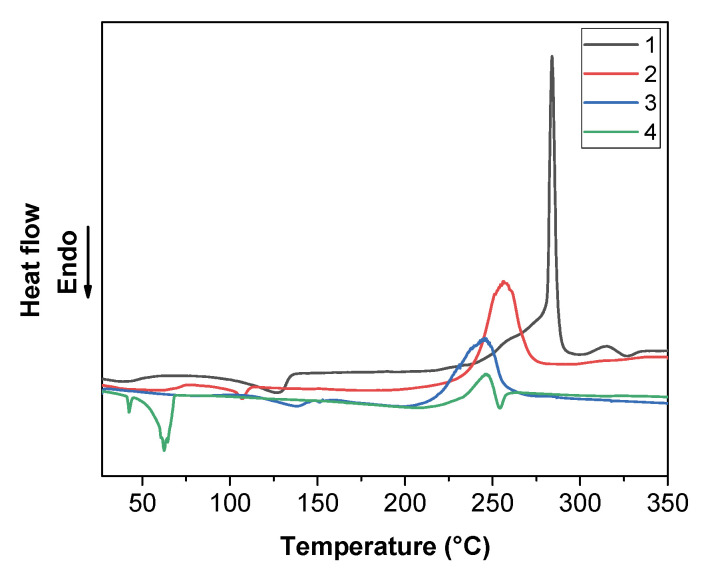
DSC profiles of [Na(hfa)]_n_ (**1**) and “Na(hfa)•glyme” (**2**, **3**, and **4**) under N_2_ flow at atmospheric pressure in the temperature range 30–350 °C.

The thermal behaviors of the as-synthesized precursors were investigated using thermogravimetric (TG) measurements (Figure 9) and differential scanning calorimetry (DSC) (Figure 10). The TG curves of adducts **3** and **4** show a single-step mass loss associated with the adducts’ vaporization, while compound **1** shows two-step mass loss and adduct **2** shows a multiple-step mass loss. Adduct **1** (Figure 9a) shows a weight loss of about 13.8% in the range 25–107 °C, which is not that straightforward and needs further analysis. The primary mass loss due to the adduct vaporization occurs in the range 215–295 °C, with a residue of 16%, which most likely corresponds to NaF formation, giving a theoretical value of 18.2%. However, this is not a drawback for its applications in liquid assisted MOCVD processes, given its solubility in common organic solvents. 

The TG curves of adducts **3** and **4** (Figure 9a) show a nearly single-step weight loss with residues of 14.2% at 274.3 °C and 9.8% at 270 °C, respectively. The adduct **2** curve (Figure 9a) shows a multi-step weight loss, also visible in the TG derivative (DTG) in Figure 9b, with a residue of about 22% at 280 °C. Interpretation of the individual steps is not straightforward, but NaF is the final residue, as assessed through EDX of the TG residue (Figure S14a). In addition, the adduct **2** powder was subjected to thermal treatment at 350 °C, and the product residue was demonstrated to be NaF through XRD measurements (Figure S14b). 

The DSC curve (Figure 10) of adduct **1** shows a small peak at 125 °C, associated with the complex melting. The DSC curve of adduct **2** presents a small endothermic peak at 107 °C due to the adduct melting, and a broad exothermic peak at 257 °C, which could be associated with its partial vaporization and decomposition.

### 2.5. Growth of NaF Nanostructures

As a proof-of-concept, we report the preliminary results of the application of the Na(hfa)•tetraglyme adduct to the fabrication of NaF nanostructures through MOCVD and sol-gel/spin-coating approaches.

**MOCVD.** The Na(hfa)•tetraglyme adduct was selected as a candidate for the MOCVD process because it shows, among the four adduct, the most suitable thermal properties, as assessed using the thermogravimetric measurements. Furthermore, the Na(hfa)•tetraglyme adduct represents a single-source precursor for both the sodium and fluorine components. Herein, for the first time, this adduct was successfully applied to the deposition of NaF thin films. 

**Figure 11:**
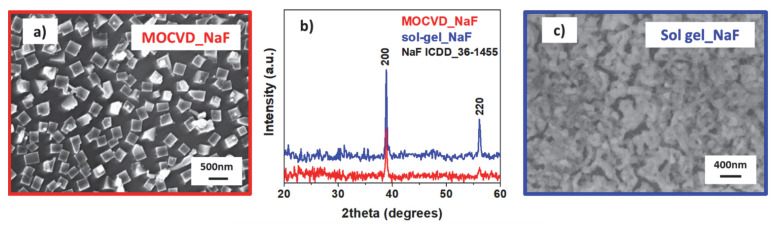
FE-SEM images and XRD patterns of the NaF nanostructures grown by MOCVD (**a**,**b**) and sol-gel/spin-coating approaches (**b**,**c**).

Phase crystallinity studies, performed with XRD analysis for both synthetic strategies applied in the present section, are reported in Figure 11b. In particular, the pattern related to the MOCVD grown nanostructures, deposited at 500 °C on the Si substrate (red line in Figure 11b), shows the formation of the polycrystalline NaF phase due to the presence of the peaks at 2θ = 38.84° and 56.09°, which may be associated with 200 and 220 reflections, respectively, of the NaF phase (ICDD N° 36–1455). 

The surface morphology of the MOCVD-grown NaF nanostructures were obtained using field emission scanning electron microscopy (FE-SEM) analysis. The FE-SEM image in Figure 11a shows the formation of NaF nanocubes of about 200 nm, uniform in shape and dimensions, homogeneously distributed over the surface.

Energy dispersive X-ray analysis (EDX) confirms the compositional purity of the NaF nanostructures. The spectrum acquired on the NaF nanocubes (Figure S15) displays the presence of a Na Kα peak at 1.04 keV and a F Kα peak at 0.677 keV. The peaks of Si Kα at 1.730 keV and O Kα at 0.560 KeV are due to the substrate. No carbon contamination was detected, thus excluding the presence of precursor impurities. 

**Sol-gel/spin-coating method.** Additionally, a combined approach incorporating the sol-gel process and spin-coating deposition was tested for the fabrication of NaF thin films starting from the Na(hfa)•tetraglyme adduct. In this case, both the clean decomposition and the high solubility of the sodium complex was tested and studied with the aim of its application in solution processes. In fact, we recently reported the successful application of this sodium precursor for the fabrication of a hexagonal lanthanide doped NaYF_4_ thin film through a combined sol-gel/spin-coating method [27,45]. The present sol-gel process is a consolidated route for the synthesis of fluoride phases, analogously to that previously reported for the synthesis of CaF_2_ thin films [27,35].

The sol was prepared starting from an ethanol solution of Na(hfa)•tetraglyme under acid catalysis, and the films were spun on Si (100) substrates through repetitive cycles and finally annealed at 400 °C in air for 1 h. Details of the procedure are reported in the experimental section.

The structural analysis of the NaF film was carried out by XRD measurement and compared with the MOCVD counterpart in Figure 11b. The pattern in blue exhibits the formation of a well crystallized layer due to the presence of the peaks at 2θ = 38.84° and 56.09°, associated with reflections of the 200 e 220 NaF phase.

Unlike with the MOCVD process, which gives rise to the formation of cubic NaF nanostructures, the morphology of the sol-gel NaF shows uniform and homogeneous films characterized by rounded grains of tens of nanometers (Figure 11c). Even in this case, the absence of impurities, such as carbon or precursor byproducts, was checked and confirmed through EDX analysis.

## 3. Materials and Methods

### 3.1. Chemical Reagents

Sodium hydroxide (NaOH, >98%) and 1,1,1,5,5,5-hexafluoro-2,4-pentanedione (H-hfa, >98%) were purchased from Strem Chemicals and used without further purification. Monoglyme (1,2-dimethoxyethane, 99.5%), diglyme (bis(2-methoxyethyl)ether, 99.5%), triglyme (2,5,8,11-tetraoxadodecane, >98%), tetraglyme (2,5,8,11,14-pentaoxapentadecane, >99%), dichloromethane (CH_2_Cl_2_, >99.5%), and n-pentane were purchased from Sigma Aldrich.

### 3.2. Synthesis and Analytical Data

#### 3.2.1. Synthesis of Na(hfa) (**1**)

The NaOH (0.367 g, 9.18 mmol) 30% excess was suspended in dichloromethane (40 mL), Hhfa (1.47 g, 7.06 mmol) was added after 10 min, and the mixture was refluxed under stirring for one hour. The excess of NaOH was filtered off. White crystals were collected after the evaporation of the solvent. The reaction yield was 88%. The melting point of the crude product was measured at around 124–126 °C using the Kofler hot-stage microscope (760 Torr). Elemental analysis (C_5_HF_6_NaO_2_): Calc: C, 26.10; H, 0.43. Found: C, 26.38; H, 0.49.

#### 3.2.2. Synthesis of “Na(hfa)•Monoglyme” (**1a**)

The NaOH (0.367 g, 9.18 mmol) 30% excess was first suspended in dichloromethane (40 mL), followed by monoglyme (0.636 g, 7.06 mmol), and, finally, Hhfa (1.47 g, 7.06 mmol) was added after 10 min, and the mixture was refluxed under stirring for one hour. The excess of NaOH was filtered off. The colorless crystals precipitated after partial evaporation of the solvent. The crystals were collected, washed with pentane, and filtered. The compound was sticky even after several cleaning steps. The reaction yield was 85%. 

#### 3.2.3. Synthesis of [Na_4_(hfa)_6_]^2−^•2[Na(Diglyme)_2_]^+^ (**2**)

The compound was synthesized from NaOH (0.367 g, 9.18 mmol), diglyme (0.636 g, 4.74 mmol), and Hhfa (1.47 g, 7.06 mmol) in dichloromethane (40 mL). The reaction yield was 80%. The melting point of the crude product was 106–108 °C (760 Torr). Elemental analysis (C_54_H_62_F_36_Na_6_O_24_): Calc: C, 33.83; H, 3.26. Found: C, 33.58; H, 3.06.

#### 3.2.4. Synthesis of Na(hfa)•Triglyme•H_2_O (**3**)

The compound was synthesized from NaOH (0.367 g, 9.18 mmol), triglyme (1.25 g, 7.06 mmol), and Hhfa (1.47 g, 7.06 mmol) in dichloromethane (40 mL). The obtained product did not crystallize and formed an oil. Elemental analysis (C_13_H_21_F_6_NaO_7_): Calc: C, 36.63; H, 4.96. Found: C, 36.12; H, 4.78.

#### 3.2.5. Synthesis of Na(hfa)•Tetraglyme (**4**)

The compound was synthesized from NaOH (0.367 g, 9.18 mmol), tetraglyme (1.57 g, 7.06 mmol), and Hhfa (1.47 g, 7.06 mmol) in dichloromethane (40 mL). The reaction yield was 87%. The crude product melts at around 63–65 °C, measured using the Kofler hot-stage microscope (760 Torr). Elemental analysis (C_15_H_23_F_6_NaO_7_): Calc: C, 39.83; H, 5.13. Found: C, 39.62; H, 5.25.

### 3.3. Methods 

#### 3.3.1. H and ^13^C NMR Spectroscopy

NMR experiments were carried out at 27 °C using a 500 MHz spectrometer (^1^H at 499.88 MHz, ^13^C-NMR at 125.7 MHz) equipped with a pulse-field gradient module (Z-axis) and a tunable 5mm Varian inverse detection probe (ID-PFG); chemical shifts (δ) are reported in ppm and are referenced to residual undeuterated solvent. 

#### 3.3.2. FT-IR Spectroscopy

Fourier Transform Infrared (FT-IR) spectra were recorded using a Jasco FT/IR-430 spectrometer with nujol mull between NaCl plates. 

#### 3.3.3. Thermogravimetric Analyses

Thermogravimetric analyses were performed using a Mettler Toledo TGA2 and STAR^e^ software. Dynamic thermal studies were carried out under purified nitrogen flow (50 sccm) at atmospheric pressure with a 5 °C min^−1^ heating rate. The weights of the samples were between 7 and 10 mg.

#### 3.3.4. Differential Scanning Calorimetry Analyses

Differential scanning calorimetry analyses were carried out using a Mettler Toledo Star System DSC 3 under purified nitrogen flow (30 sccm) at atmospheric pressure with a 5 °C/min heating rate. The weights of the samples were between 5 and 8 mg. Melting points were taken on tiny single crystals using a Kofler hot-stage microscope.

#### 3.3.5. X-ray Crystallographic Procedures

Intensity data from single crystals of adduct **1** and adduct **2** were collected at 100 K with a Bruker APEX-II CCD using the Cu-Kα radiation (λ = 1.54184 Å). Data collections were performed with the program CrysAlis CCD [46]. Data reductions were carried out with the program CrysAlis RED [46,47]. Finally, absorption corrections were performed with the program ABSPACK in CrysAlis RED. Structures were solved using the SIR-2004 package [47], and subsequently refined on the F^2^ values by the full-matrix least-squares program SHELXL-2013 [48]. All the non-hydrogen atoms of the two structures were refined using anisotropic thermal parameters. In contrast, the hydrogen atoms were set in a calculated position and refined in agreement with the carbon atoms to which they were bound. The quality of the crystals of **1** was not excellent. As a consequence, several crystals were tested, and the data reported in the present work are those obtained for the best of them. Similarly, several crystals of adduct **2** were tested, and all turned out to be twin-crystals. The data reported in the article are those obtained for the best single crystal available; this crystal was composed of two twins whose data were separately reduced using CrysAlis RED. Geometrical calculations were performed by PARST97 [49], and molecular plots were produced by the program Mercury (v3.7) [50]. Crystallographic data and refinement parameters are reported in Table S3. 

Deposition numbers 2107734 (for [Na(hfa)]_n_, **1**), 2107735 (for [Na_4_(hfa)_6_]^2−^•2[Na(diglyme)_2_]^+^, **2**) contain the supplementary crystallographic data for this paper. These data are provided free of charge by the joint CCDC/FIZ Karlsruhe deposition service.

### 3.4. Synthesis of NaF Nanostructures

The MOCVD depositions were carried out on a Si substrate in a low-pressure, hot wall MOCVD reactor under O_2_ flow (150 sccm) as reacting gas and Ar flow (150 sccm) as carrier gas. The substrate temperature was maintained at 500 °C. The precursor source temperature was fixed at the value of 110 °C. The sol was prepared from ethanol solution under acid catalysis according to the following molar ratio: 2 Na(hfa)tetraglyme:43.5 C_2_H_5_OH:1.5 H_2_O:0.8 CF_3_COOH. The sol was stirred at 60 °C for 20 h and successively spin-coated on Si (100) substrates. The spin coating process was carried out in four subsequent steps. In each step, 0.2 mL of the gel was spun for 60 s on the Si substrate, and after each step the sample was heated at 400 °C in air for 10 min. A final annealing treatment was performed for 1 h at 400 °C in air.

## 4. Conclusions

In summary, a one-pot synthetic method was successfully applied for the production, in high yield, of sodium precursors, using β-diketonate ligands to counter-balance the charge of the cation, and different polyethers to complete the coordination sphere. Complex **1** and the glyme adducts **2** and **4** were solid, whereas the adduct **3** was a liquid. Depending on the nature of the glyme, the precursors demonstrated interesting structural motifs, from the complex ionic structure for the diglyme adduct, [Na_4_(hfa)_6_]^2−^•2[Na(diglyme)_2_]^+^, to the monomeric nature for the tetraglyme complex, Na(hfa)•tetraglyme, as assessed through single-crystal X-ray diffraction studies. FT-IR spectra confirmed the coordination of the hfa anion for all the adducts, and of the glyme ligands for adducts **2**, **3**, and **4**, thus confirming the formation of the liquid adduct **3** of the type Na(hfa)•triglyme•H_2_O. The thermal analyses revealed a single-step mass loss for adducts **3** and **4**, with residues of 14.2% and 9.8%, respectively. Adduct **1** showed a two-step mass loss with a residue of 16%, and adduct **2** showed a multiple-step mass loss. 

Functional validation of one of these precursors, the Na(hfa)•tetraglyme, was assessed through applications to the synthesis of NaF films using two different challenging approaches, namely MOCVD and sol-gel/spin-coating.

## Figures and Tables

**Table 1:** ^1^H and ^13^C NMR chemical shift data (ppm) of complexes **1**–**4**.

^1^H NMR			^13^C NMR			
Complex	CO-C*H=*C-O^−^	Polyether *^a^*	CO-*C*H=C-O^−^	*C*O-CH=*C*-O^−^	*C*F_3_	Polyether *^a^*
1	5.62 (s)	-	85.50	175.35 (q, ^2^J_CF_ = 30.7 Hz)	119.16 (q, ^1^J_CF_ = 290.3 Hz)	-
2	5.65 (s, 6H)	a: 3.33 (s, 24H) b, 3.50 (m, 16H) c: 3.58 (m, 16 H)	85.44	175.28 (q, ^2^J_CF_ = 30.6 Hz)	119.17 (q, ^1^J_CF_ = 290.1 Hz)	a: 58.83 b: 70.79 c: 72.45
3	5.65 (s, 1H)	a: 3.33 (s, 6H) b: 3.51(m, 4H) c, d: 3.59 (m, 8H)	84.54	174.35 (q, ^2^J_CF_ = 30.7 Hz)	118.25 (q, ^1^J_CF_ = 290.1 Hz)	a: 57.99 b, c: 69.58 d: 71.22
4	5.63 (s, 1H)	a: 3.3a (s, 6H) b: 3.51 (m, 4H) c, d, e: 3.59 (m, 12H)	85.43	175.22 (q, ^2^J_CF_ = 30.7 Hz)	119.19 (q, ^1^J_CF_ = 290.2 Hz)	a: 58.96 b: 70.32 c, d: 70.38 e: 72.13

*^a^* The following notation has been used for the polyether moieties: diglyme (CH_3_^a^-O-CH_2_^b^CH_2_^c^)_2_-O, triglyme (CH_3_^a^-O-CH_2_^b^-CH_2_^c^-O-CH_2_^d^-)_2_, and tetraglyme (CH_3_^a^-O-CH_2_^b^-CH_2_^c^-O-CH_2_^d^-CH_2_^e^-)_2_-O.

## Data Availability

Not applicable.

## Supplementary Materials

The following supporting information can be downloaded at: https://www.mdpi.com/article/10.3390/molecules27196282/s1, Table S1**:** Bond lengths in **1***;* Table S2: Selected bond lengths in **2**; Table S3: Crystallographic data and refinement parameters for **1** and **2**; Figure S1: Scheme of the bridging of the hfa ligand in **1**; Figures S2–S13: ^1^H-NMR and ^13^C-NMR spectra of adducts **1**–**4**; Figure S14: EDX and XRD of the residue of adduct **2**; Figure S15: EDX spectrum of the NaF structures.
