# Supplementary material for: Sodium β-Diketonate Glyme Adducts as Precursors for Fluoride Phases: Synthesis, Characterization and Functional Validation
Source: Molecules. 2022 Sep 23;27(19):6282. doi: 10.3390/molecules27196282 (PMC9571527; doi:10.3390/molecules27196282)
Supplement: Supplementary file 1 [file molecules-27-06282-s001.zip › Supporting/Supporting information.pdf]

Supporting Information

Sodium β-diketonate glyme adducts as precursors for fluoride phases: synthesis, characterization and functional validation

Nishant Peddagopu,[a] Anna L. Pellegrino,[a] Carmela Bonaccorso,[b] Patrizia Rossi,[c] Paola Paoli,[c] and Graziella Malandrino[a,*]

[a] Dipartimento di Scienze Chimiche, University of Catania and INSTM UdR Catania, V.le A. Doria 6, 95125 Catania, Italy
[b] Dipartimento di Scienze Chimiche, Università di Catania, V.le Andrea Doria 6, 95125 Catania, Italy.
[c] Dipartimento di Ingegneria Industriale, Università di Firenze, Via Santa Marta 3, 50139 Firenze, Italy

Table of contents

**Figure S1**	Scheme of the bridging of the hfa ligand in 1.	*2*
**Table S1**	Na-X distances in compound **1**.	*3*
**Table S2**	Selected bond lengths in **2**.	*4*
**Table S3**	Crystallographic data and refinement parameters for **1** and **2**.	*6*
**Figure S2**	^{1}H-NMR spectrum of **1** (solvent CD_3CN).	*7*
**Figure S3**	^{13}C-NMR spectrum of **1** (solvent CD_3CN).	*8*
**Figure S4**	^{1}H-NMR spectrum of **2** (solvent CD_3CN).	*9*
**Figure S5**	^{13}C-NMR spectrum of **2** (solvent CD_3CN).	*10*
**Figure S6**	^{1}H-NMR spectrum of **3** (solvent CD_3CN).	*11*
**Figure S7**	^{1}H-NMR spectrum of **3** (solvent $CDCl_3$).	*12*
**Figure S8**	^{13}C-NMR spectrum of **3** (solvent CD_3CN).	*13*
**Figure S9**	^{13}C-NMR spectrum of **3** (solvent $CDCl_3$).	*14*
**Figure S10**	^{1}H-NMR spectrum of **4** (solvent CD_3CN).	*15*
**Figure S11**	^{1}H-NMR spectrum of **4** (solvent $CDCl_3$).	*16*
**Figure S12**	^{13}C-NMR spectrum of **4** (solvent CD_3CN).	*17*
**Figure S13**	^{13}C-NMR spectrum of **4** (solvent $CDCl_3$).	*18*
**Figure S14**	EDX spectrum and XRD pattern of the NaF formed after thermal treatment of **2.**	*19*
**Figure S15**	EDX spectrum of the NaF structures.	*20*

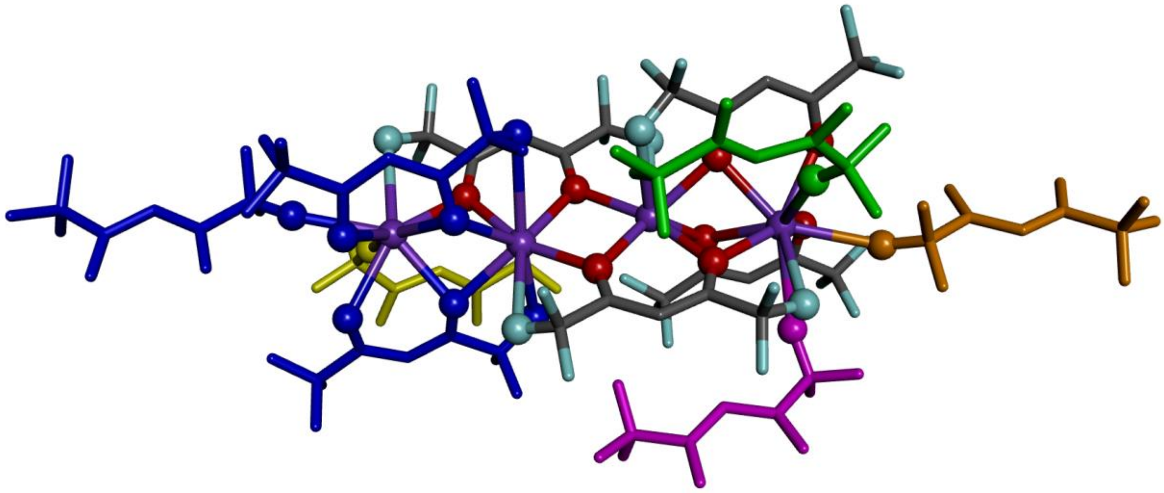

Figure S1. Bridging scheme of the hfa ligand in **1.** For the sake of clarity, hydrogen atoms have been omitted. Legenda: orange: x,y,z+1; green: y,-x+1,-z+1; magenta: -y+0.5, x+0.5, -z+1.5; blue: x, y, z-1; yellow: y-0.5, -x+0.5, -z+0.5

Table S1. Na-X distances in compound **1**.

Na-X	distance (Å)	Symmetry operation
Na1-O1	2.463(8)	
Na1-O2	2.446(8)	
Na1-O3	2.442(8)	
Na1-O4	2.699(8)	
Na1-O5	2.337(8)	
Na1-F14a	2.861(8)	
Na1-F10b	2.844(8)	-y+1/2,x+1/2,-z+3
Na1-F15a	2.695(8)	x,y,z+1
Na1-F20b	2.742(7)	y,-x+1,-z+1
Na2-O2	2.346(8)	
Na2-O4	2.332(8)	
Na2-O5	2.351(8)	
Na2-O6	2.324(8)	
Na2-O7	2.330(8)	
Na2-F5a	2.76(1)	
Na2-F19a	2.864(8)	
Na3-O6	2.335(8)	
Na3-O7	2.346(8)	
Na3-O8	2.330(8)	
Na3-F15a	2.909(8)	
Na3-O1	2.394(8)	x,y,z-1
Na3-F4b	3.01(1)	x,y,z-1
Na3-F9a	2.682(9)	x,y,z-1
Na4-O8	2.323(8)	
Na4-F20a	2.835(8)	
Na4-O1	2.605(8)	x,y,z-1
Na4-O2	2.444(8)	x,y,z-1
Na4-O3	2.434(8)	x,y,z-1
Na4-O4	2.495(8)	x,y,z-1
Na4-F14b	2.425(7)	y-1/2,-x+1/2,-z+1/2
Na4-F19a	2.661(8)	x,y,z-1

Table S2. Selected bond lengths in **2**.

Na-X	distance (Å)
Na1···Na2	3.192(5)
Na2···Na3	3.549(5)
Na3···Na4	3.174(4)
Na1-O1	2.255(7)
Na1-O2	2.364(8)
Na1-O3	2.288(8)
Na1-O4	2.349(6)
Na1-O5	2.306(8)
Na1-F14a	2.945(7)
Na2-O2	2.406(7)
Na2-O4	2.345(8)
Na2-O5	2.370(6)
Na2-O6	2.417(7)
Na2-O7	2.373(6)
Na2-F19a	2.689(6)
Na3-O6	2.343(6)
Na3-O7	2.397(7)
Na3-O8	2.373(6)
Na3-F15a	2.700(7)
Na3-O9	2.355(7)
Na3-O11	2.342(8)
Na4-O8	2.310(7)
Na4-O9	2.351(8)
Na4-O10	2.272(7)
Na4-O11	2.346(6)
Na4-O12	2.274(9)
Na5-O13	2.407(8)
Na5-O14	2.386(7)
Na5-O15	2.393(8)
Na5-O16	2.378(9)
Na5-O17	2.401(8)

Na5-O18	2.382(7)
Na6-O19	2.401(7)
Na6-O20	2.371(8)
Na6-O21	2.377(6)
Na6-O22	2.373(9)
Na6-O23	2.409(7)
Na6-O24	2.357(8)

Table S3. Crystallographic data and refinement parameters for **1** and **2**.

	**1**	**2**
Empirical formula	$[Na(hfa)]_n$	$[Na_4(hfa)_6]^{2-}$•2$[Na(diglyme)_2]^+$
Formula weight	230.05	1916.97
Temperature (K)	100	100
Wavelength (Å)	1.54184	1.54184
Crystal system, space group	tetrahedral, I-4	Triclinic, P-1
Unit cell dimensions (Å, °)	a = 24.4916(7)<br>b = 24.4916(7)<br>c = 9.8714(3)	a =11.460(1) α = 83.860(4)<br>b = 17.019(1) β = 87.651(5)<br>c = 21.816(2) γ = 71.840(4)
Volume ($Å^3$)	5921(2)	4019.6(6)
Z, D_c (mg/cm^3)	8, 2.064	2, 1.584
μ (mm^{-1})	2.803	1.839
F(000)	3584	1936
θ range (°)	2.551-68.403	2.037-68.696
Reflections collected / unique	18361 / 5247	100935 / 14654
Data / parameters	5247 / 505	14654 / 1082
Goodness-of-fit on F^2	0.993	1.927
Final R indices [$I>2\sigma(I)$]	R1 = 0.0821, wR2 = 0.2238	R1 = 0.1584, wR2 = 0.4446
R indices (all data)	R1 = 0.1087, wR2 = 0.2541	R1 = 0.1751, wR2 = 0.4627

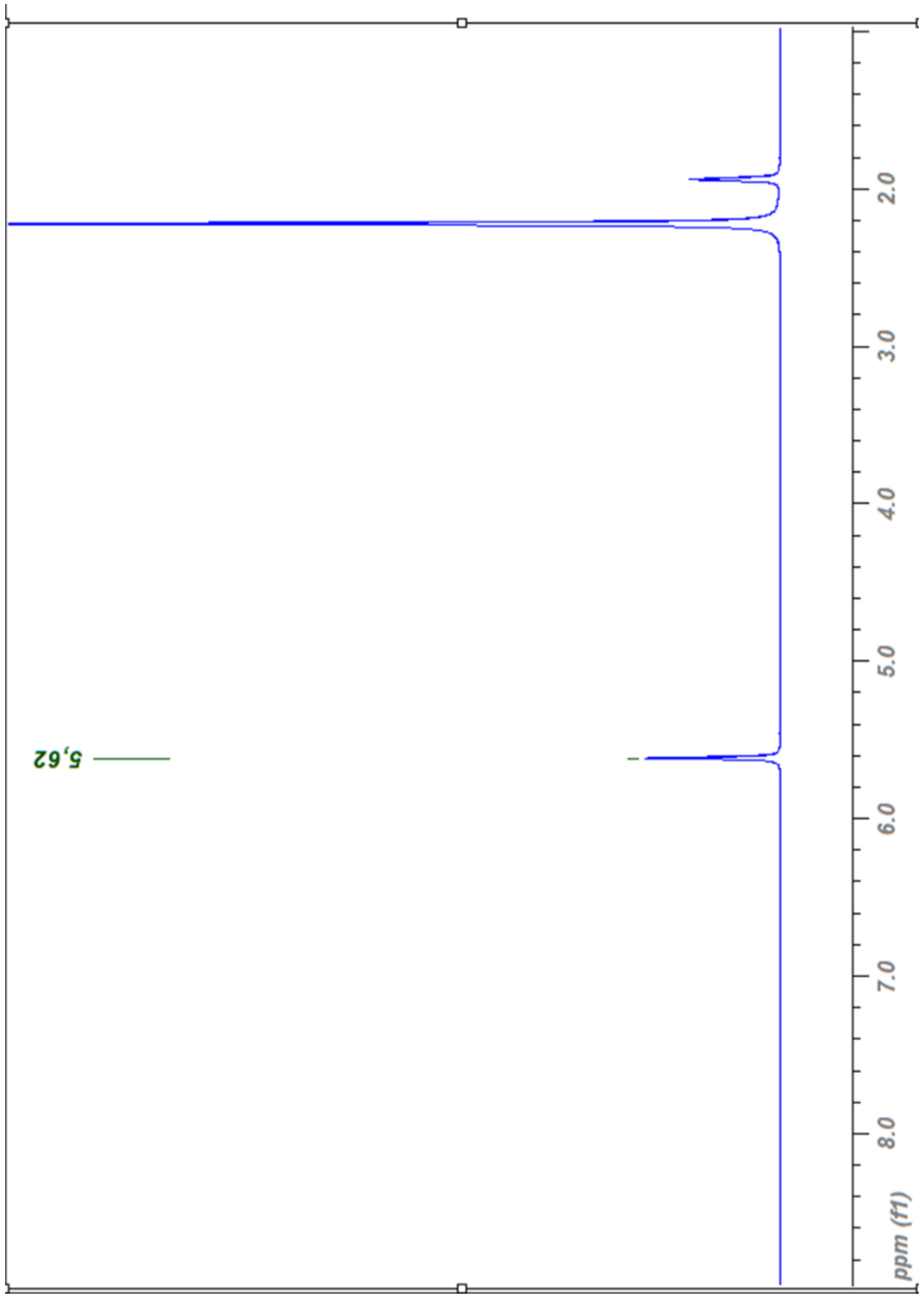

Figure S2. ^{1}H-NMR spectrum of **1** (CD_3CN, 500 MHz, 27 °C)

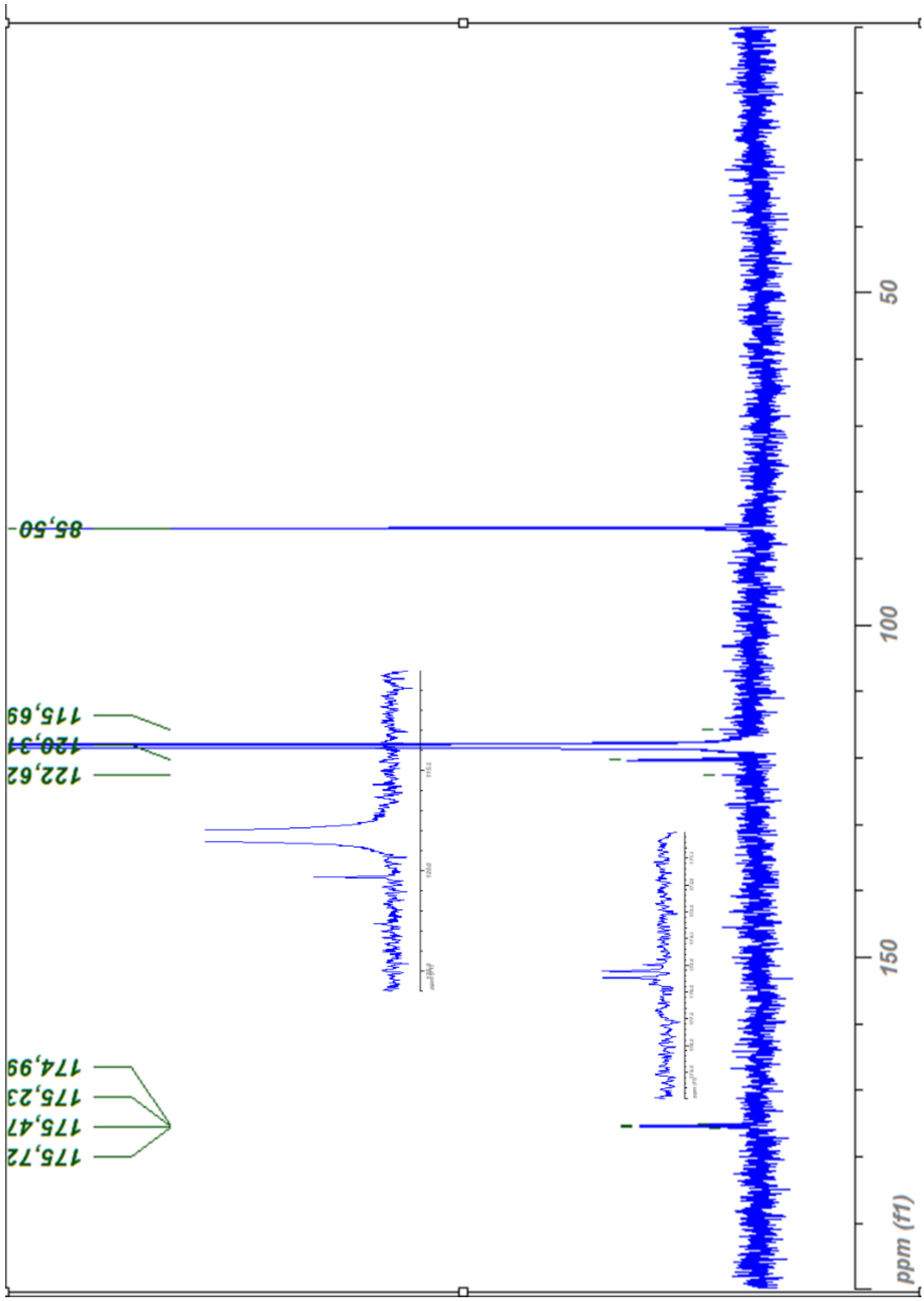

Figure S3. ^{13}C-NMR spectrum of **1** (CD_3CN, 125 MHz, 27 °C)

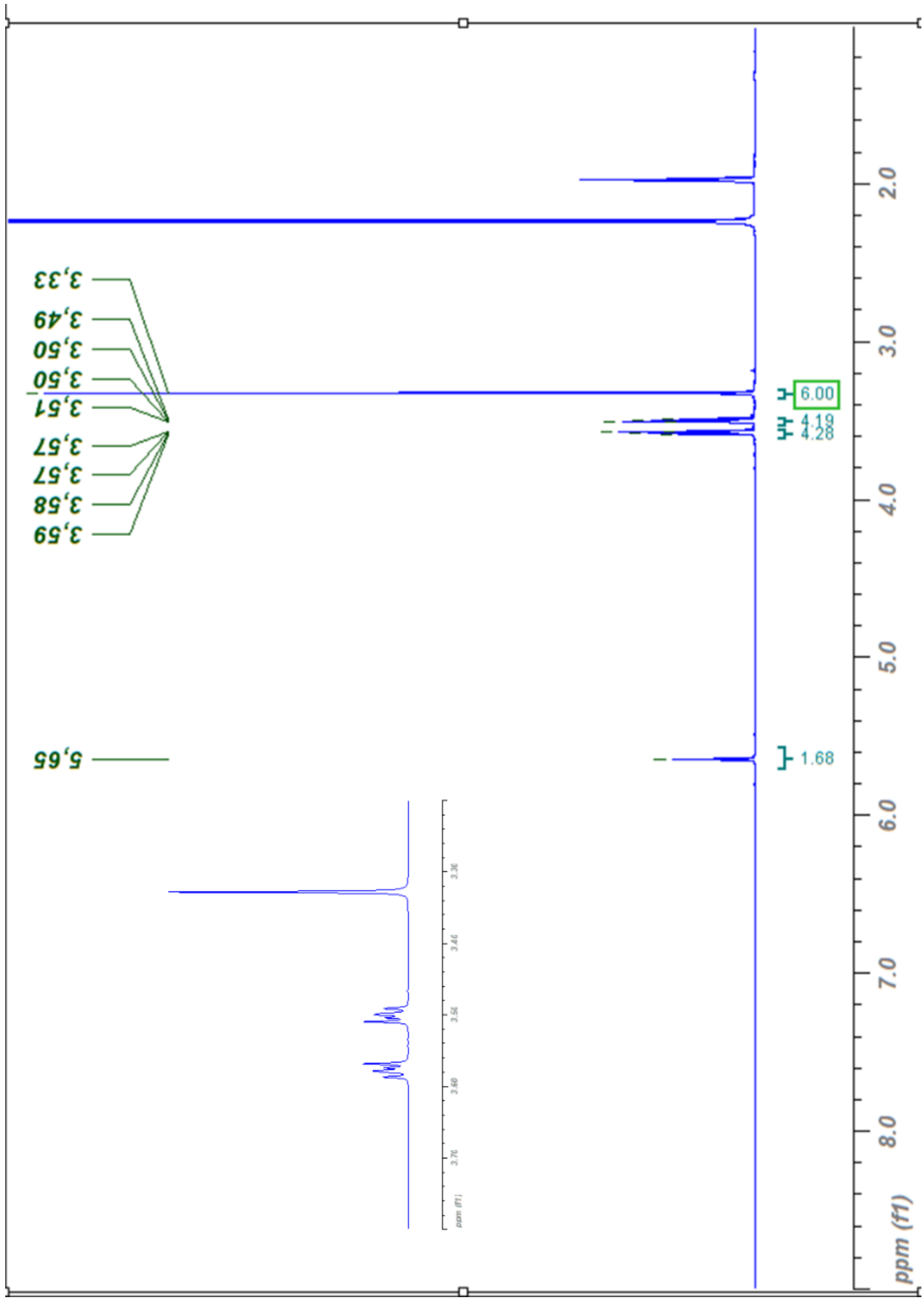

Figure. S4. ^{1}H-NMR spectrum of **2** (CD_3CN, 500 MHz, 27 °C)

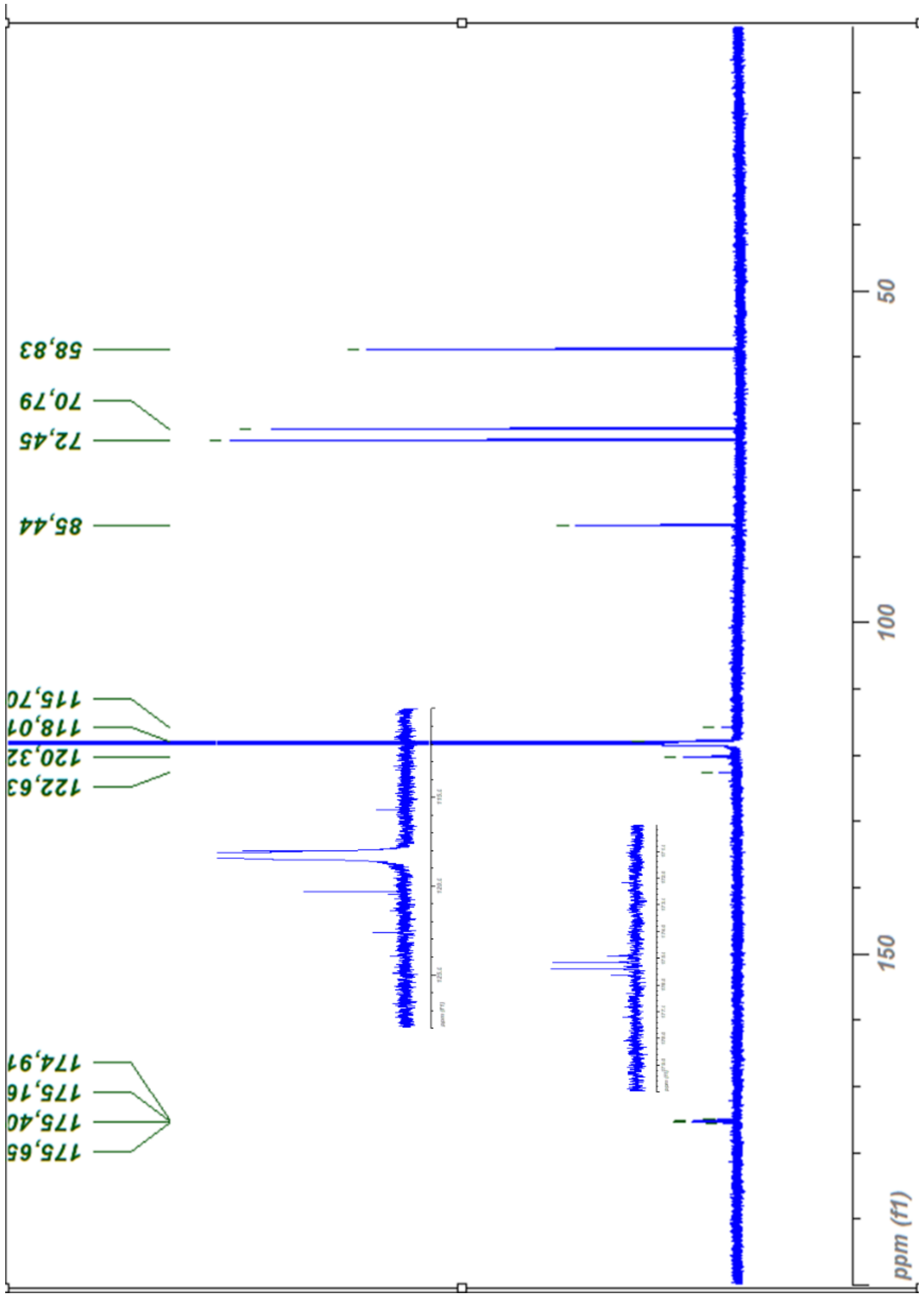

Figure S5. ^{13}C-NMR spectrum of **2** (CD_3CN, 125 MHz, 27 °C)

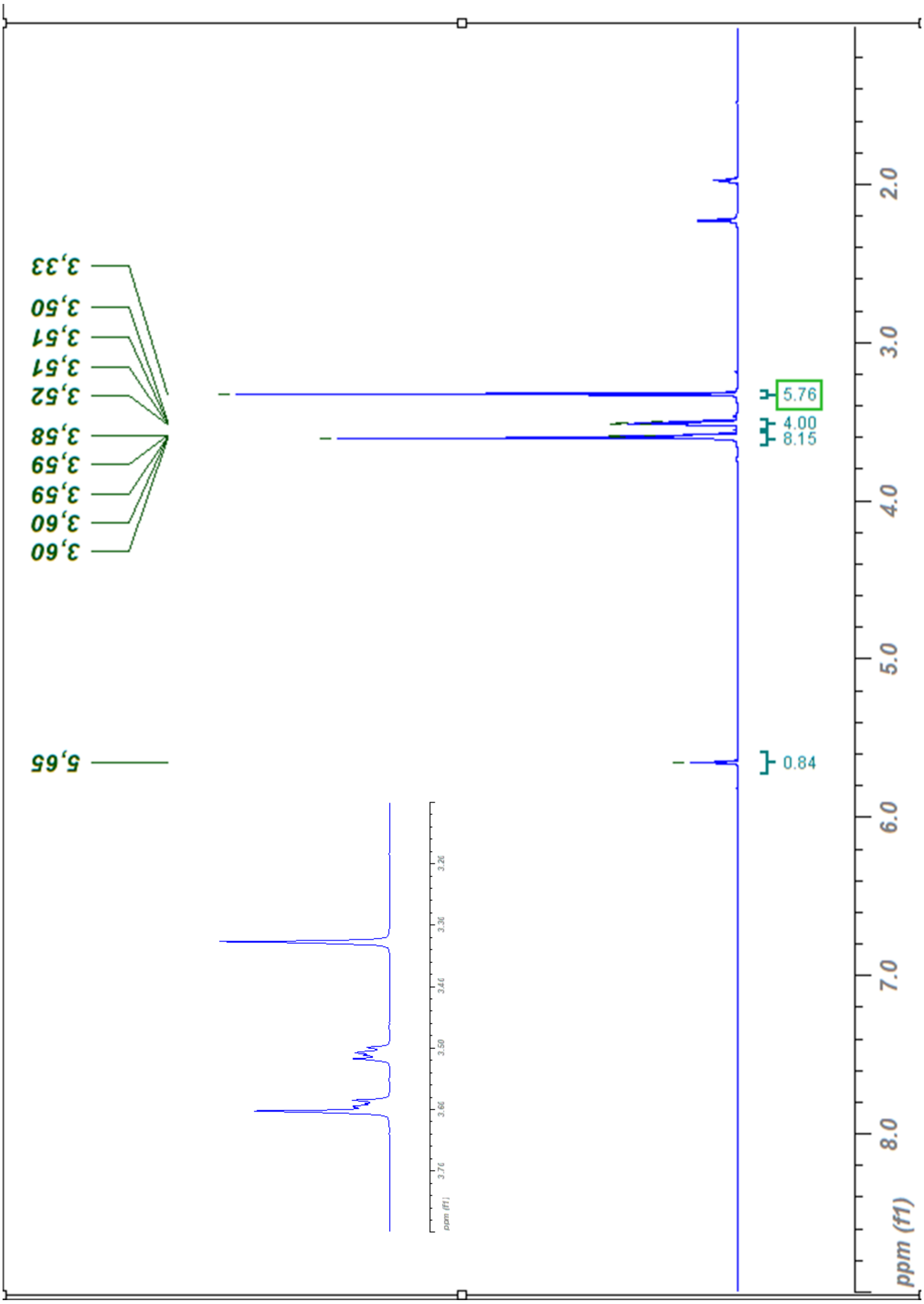

Figure. S6. ^{1}H-NMR spectrum of **3** (CD_3CN, 500 MHz, 27 °C)

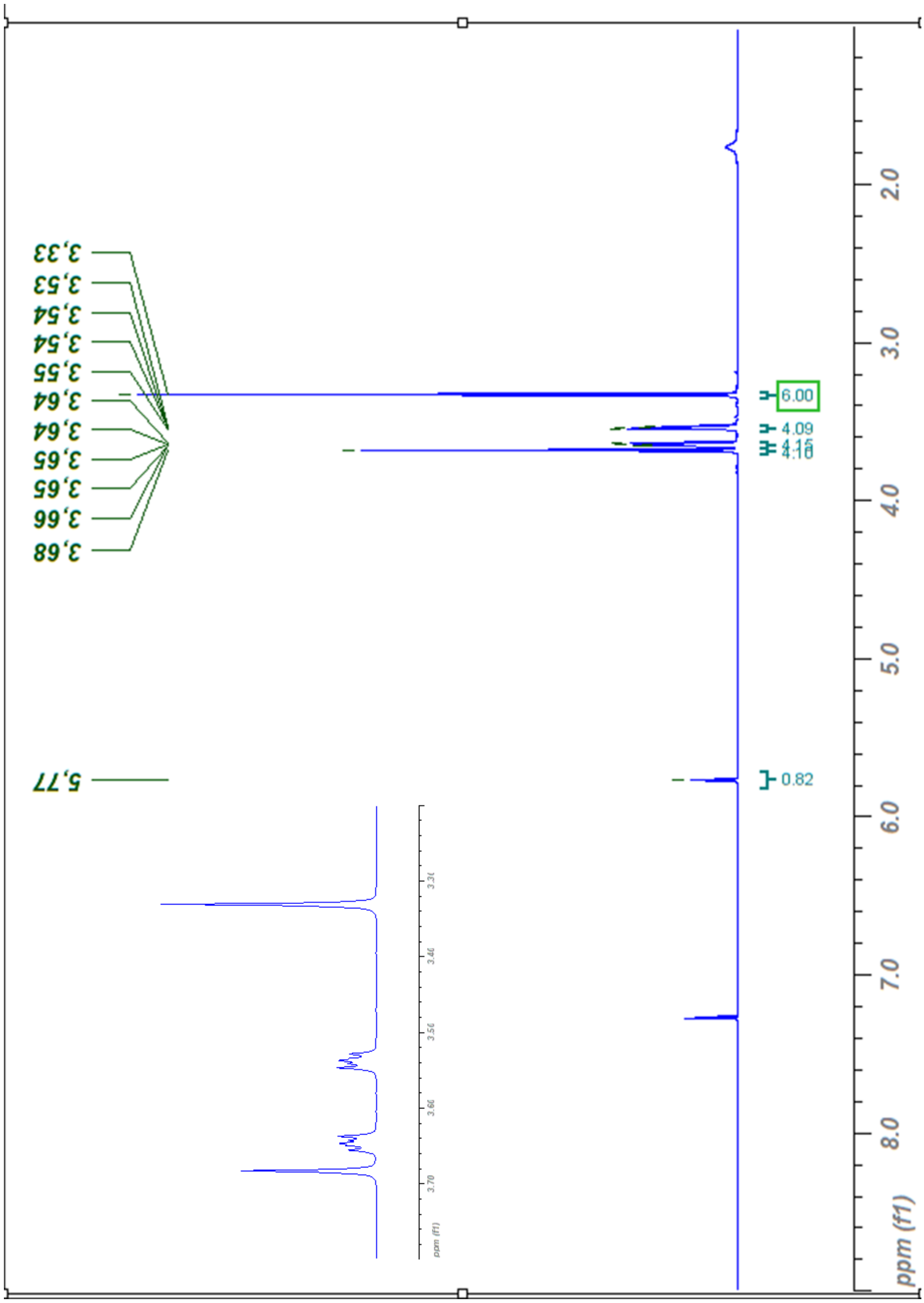

Figure. S7. ^{1}H-NMR spectrum of **3** ($CDCl_3$, 500 MHz, 27 °C)

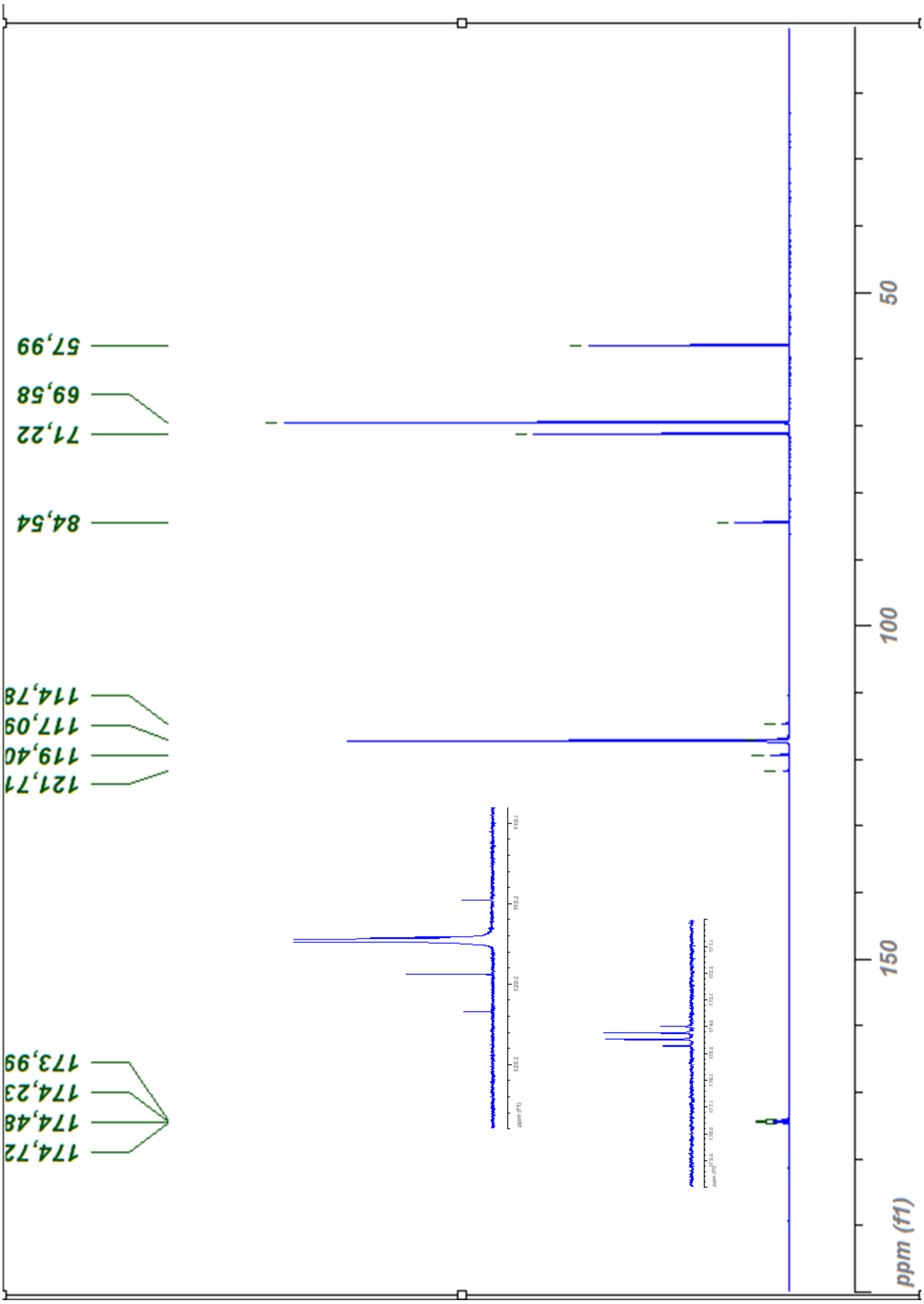

Figure. S8. ^{13}C-NMR spectrum of **3** (CD_3CN, 125 MHz, 27 °C)

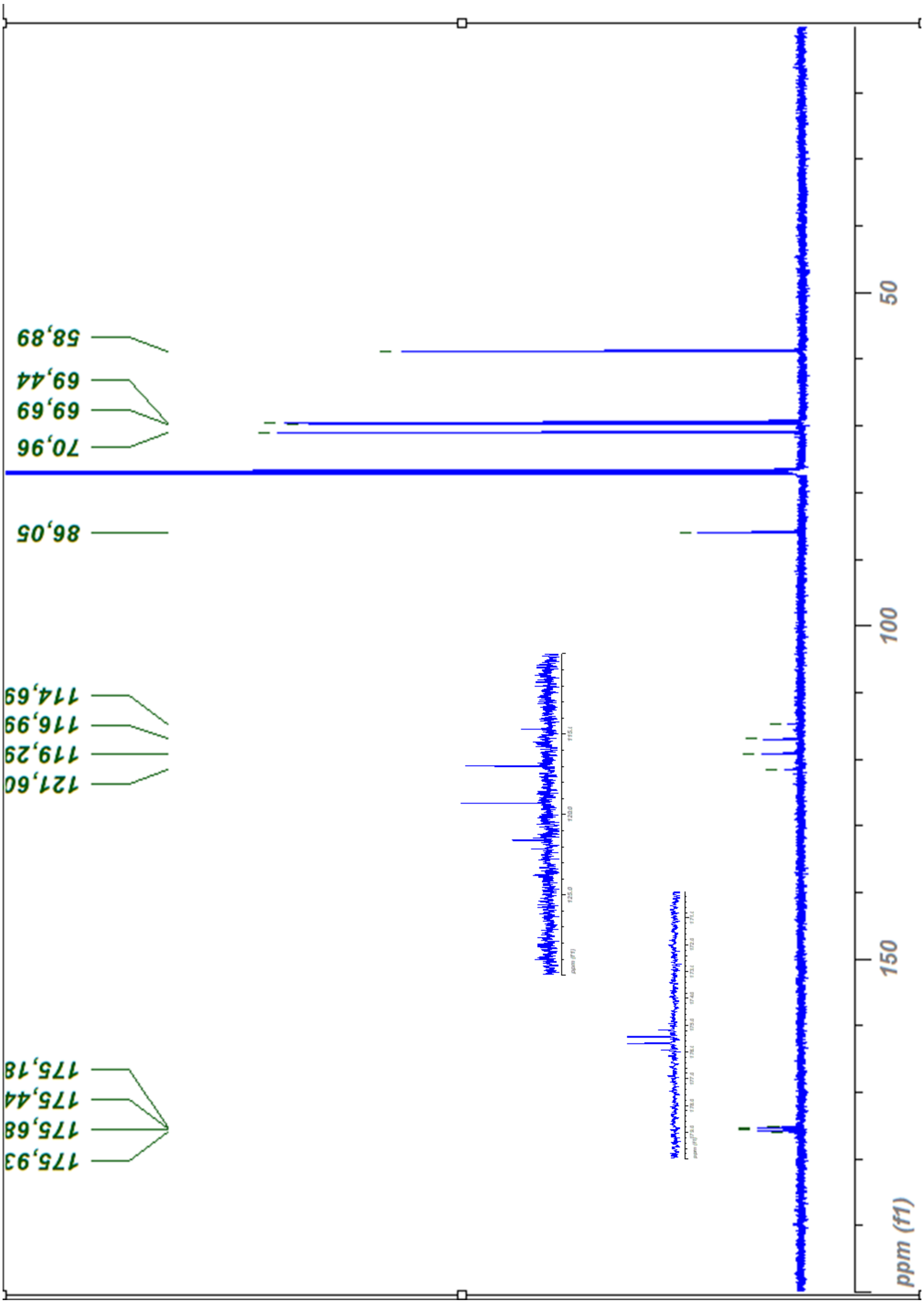

Figure. S9. ^{13}C-NMR spectrum of **3** ($CDCl_3$, 125 MHz, 27 °C)

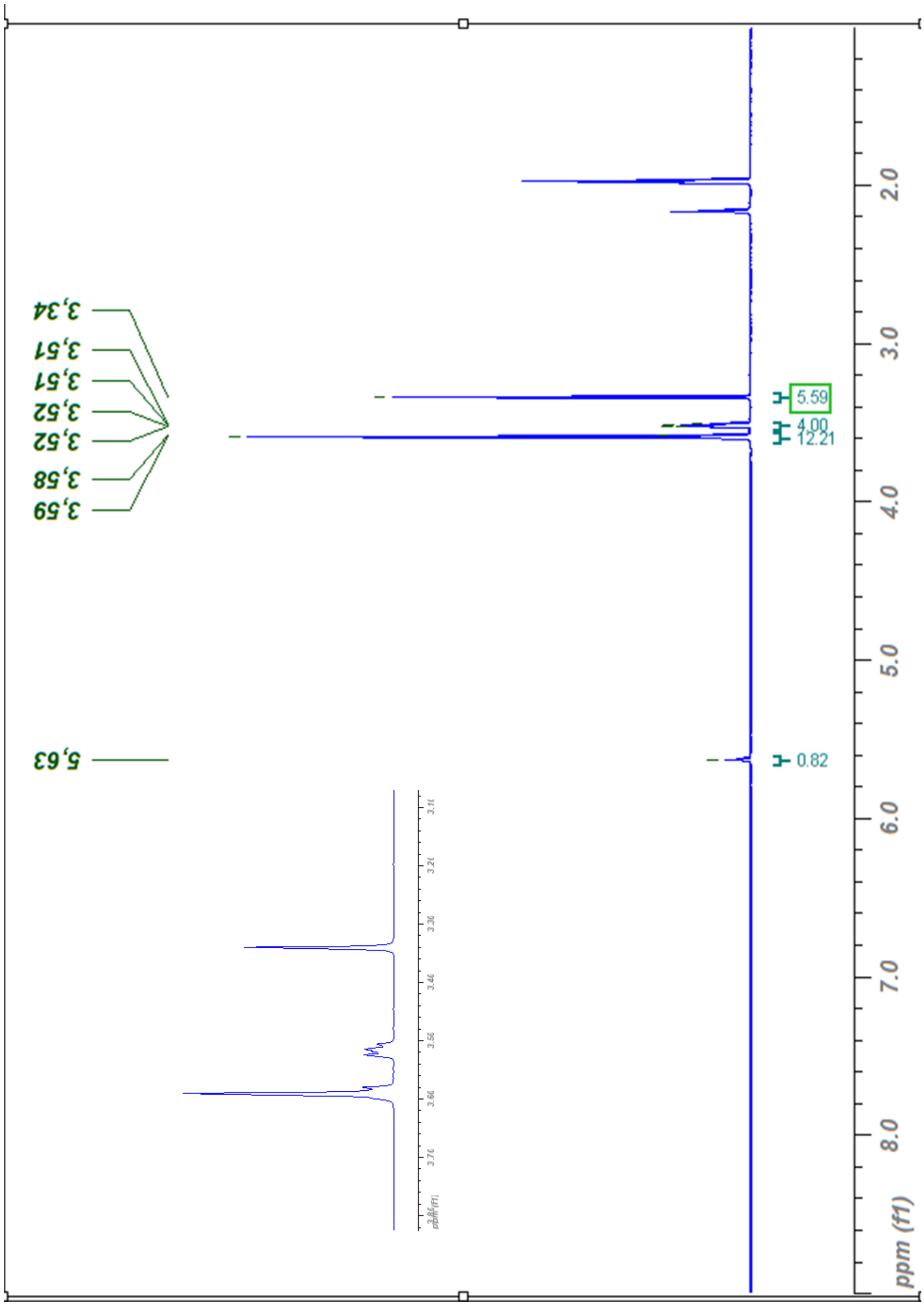

Figure. S10. ^{1}H-NMR spectrum of **4** (CD_3CN, 500 MHz, 27 °C)

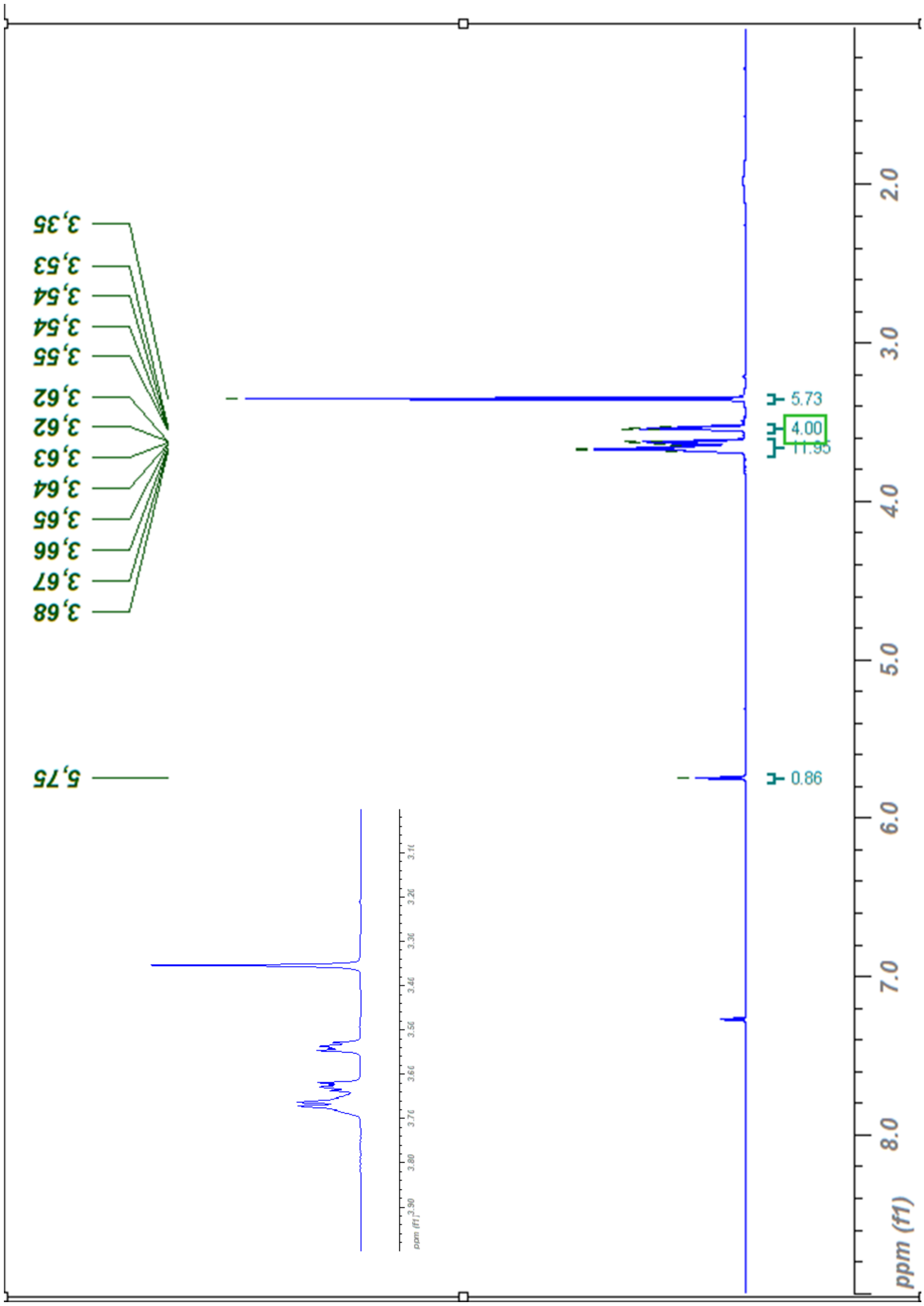

Figure. S11. ^{1}H-NMR spectrum of **4** ($CDCl_3$, 500 MHz, 27 °C)

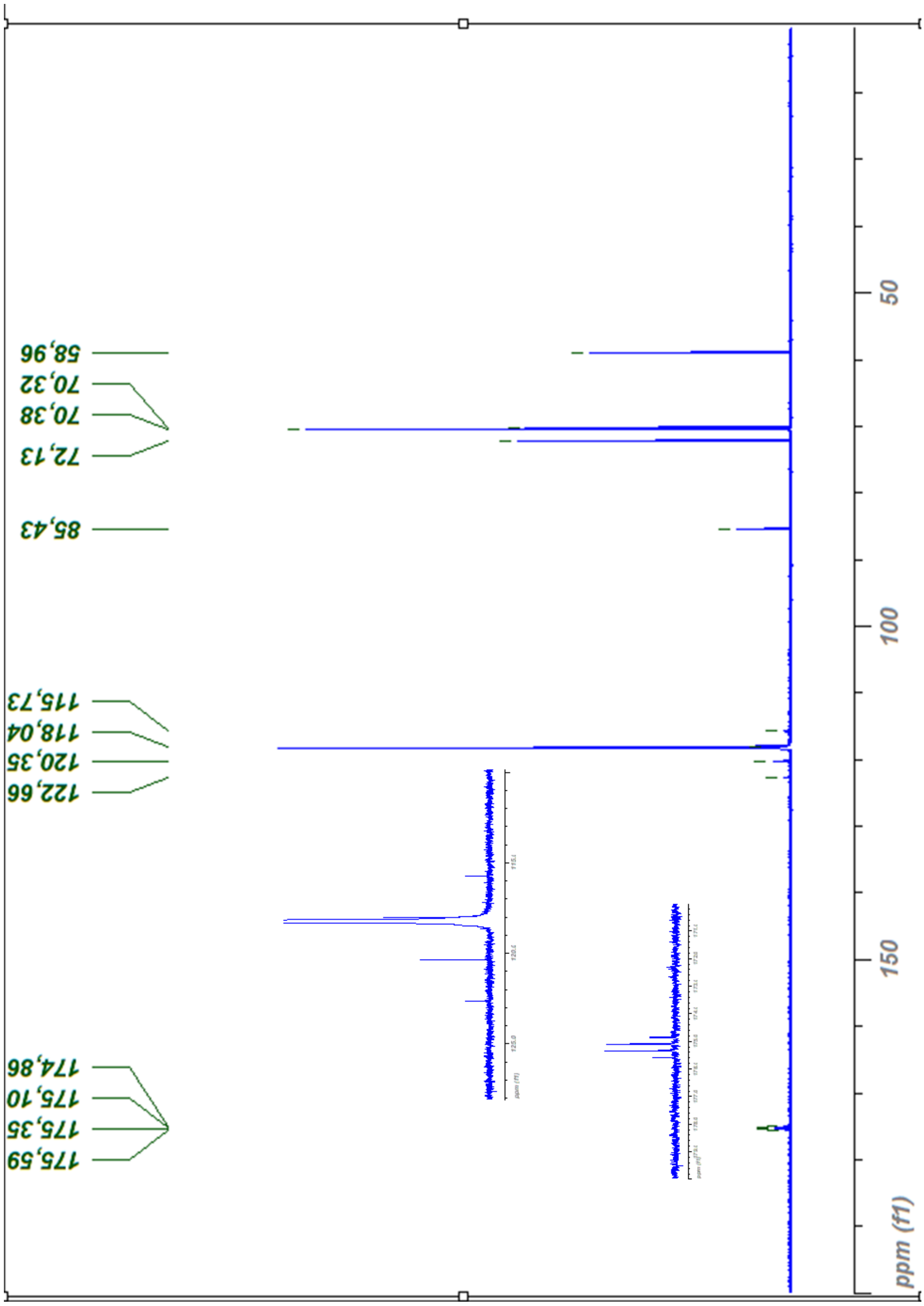

Figure. S12. ^{13}C-NMR spectrum of **4** (CD_3CN, 125 MHz, 27 °C)

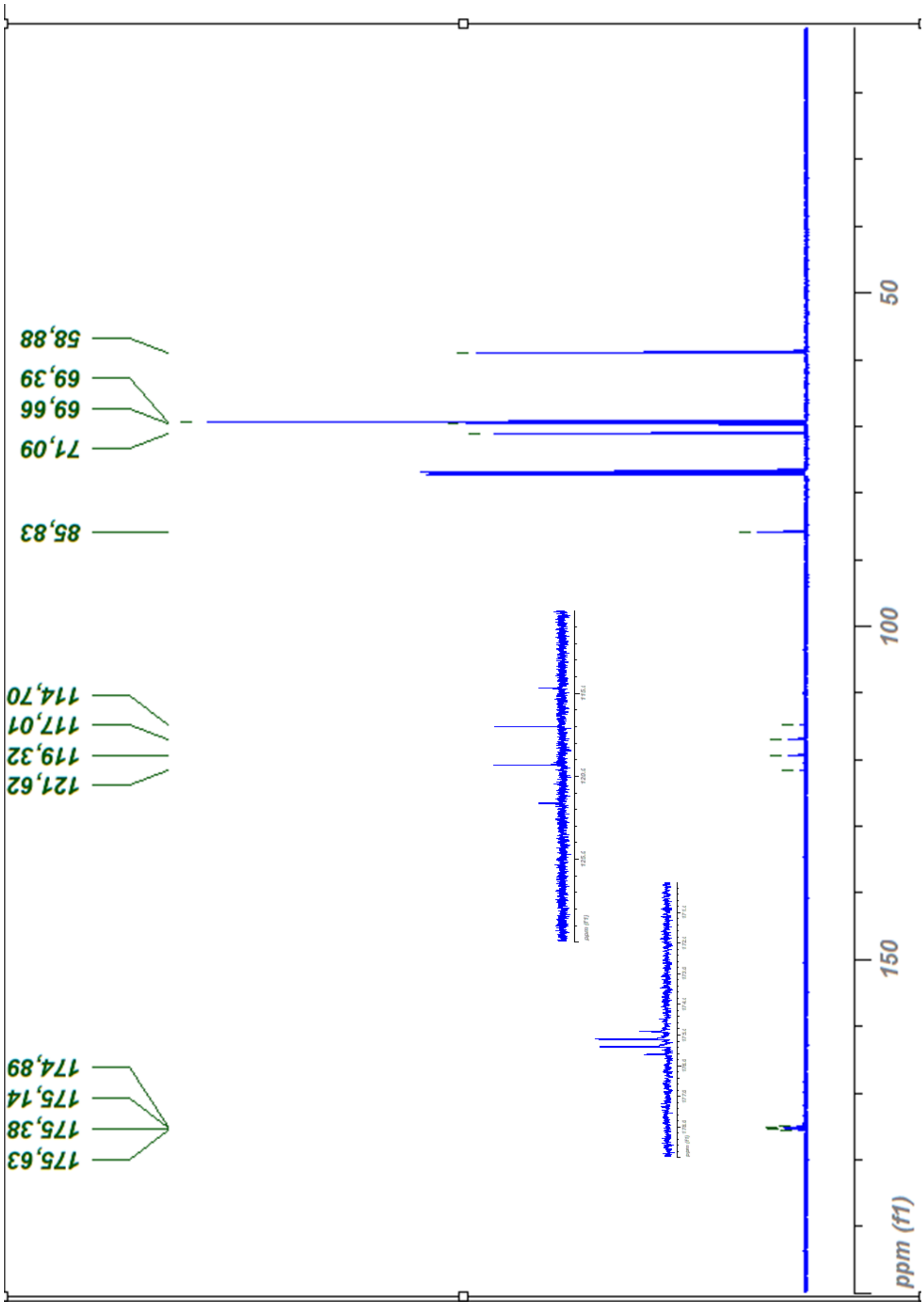

Figure S13. ^{13}C-NMR spectrum of **4** ($CDCl_3$, 125 MHz, 27 °C)

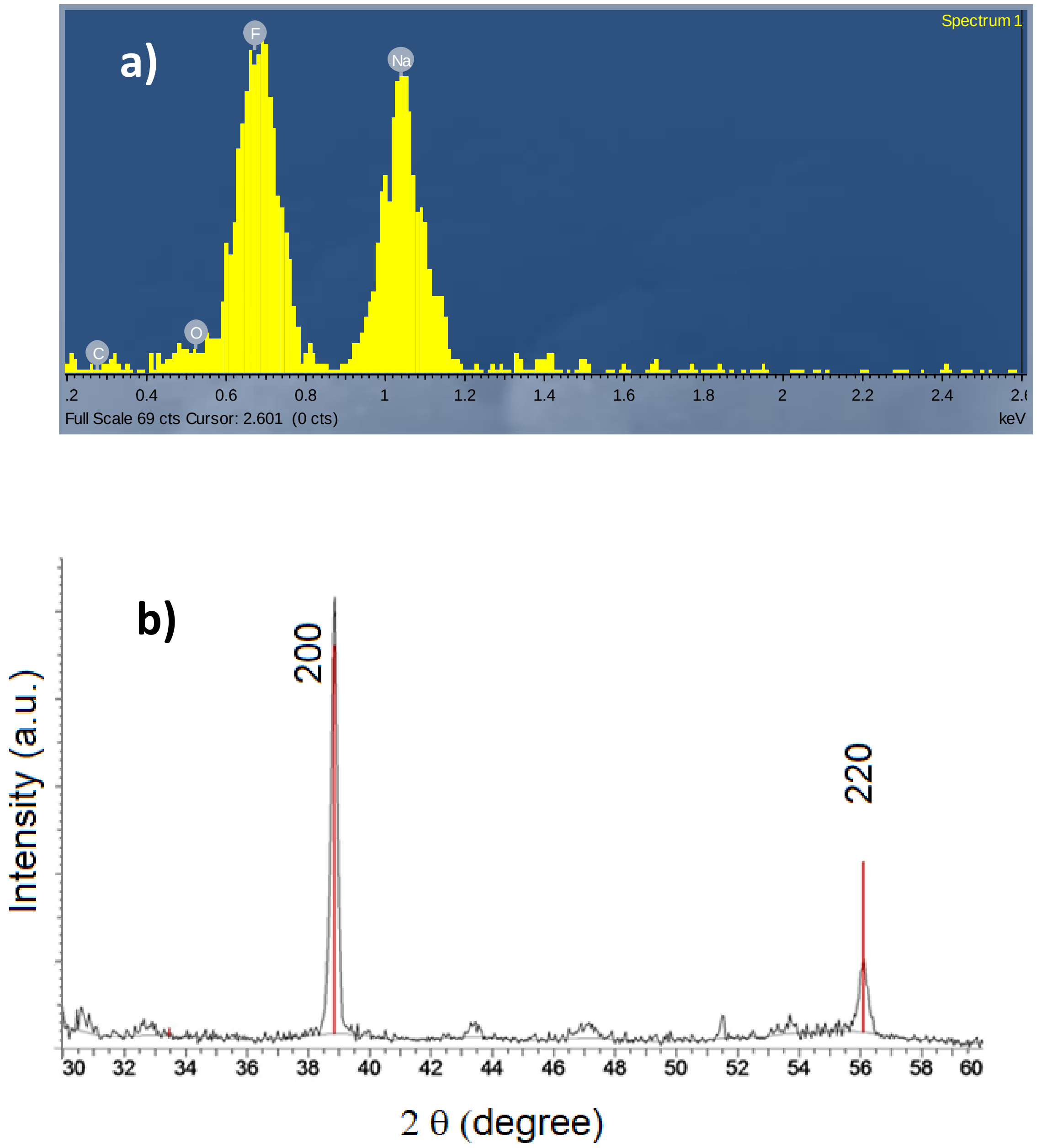

Figure S14. a) EDX spectrum of the TGA residue of the adduct **2**; b) XRD pattern of the powder obtained after treatment at 350 °C of the adduct **2**.

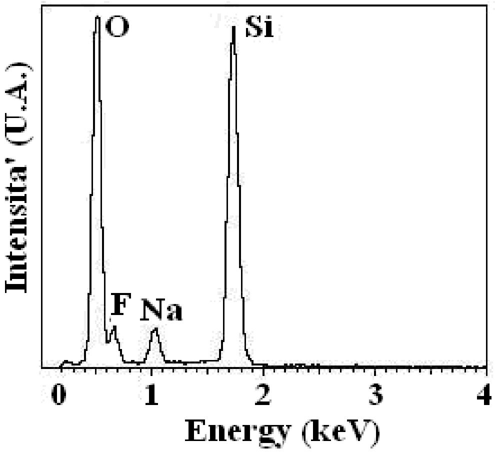

Figure S15. EDX spectrum of the NaF nanocubes deposited through MOCVD.
