# Supplementary material for: Sodium β-Diketonate Glyme Adducts as Precursors for Fluoride Phases: Synthesis, Characterization and Functional Validation
Source: Molecules. 2022 Sep 23;27(19):6282. doi: 10.3390/molecules27196282 (PMC9571527; doi:10.3390/molecules27196282)

checkCIF/PLATON report

Structure factors have been supplied for datablock(s) 1

THIS REPORT IS FOR GUIDANCE ONLY. IF USED AS PART OF A REVIEW PROCEDURE FOR PUBLICATION, IT SHOULD NOT REPLACE THE EXPERTISE OF AN EXPERIENCED CRYSTALLOGRAPHIC REFEREE.

No syntax errors found. CIF dictionary Interpreting this report

Datablock: 1

```
Bond precision:   C-C = 0.0144 A          Wavelength=1.54178

Cell:              a=24.4916(7)      b=24.4916(7)      c=9.8714(3)
                   alpha=90          beta=90           gamma=90
Temperature:       100 K

                 Calculated                    Reported
Volume           5921.3(4)                     5921.2(4)
Space group      I -4                          I -4
Hall group       I -4                          I -4
Moiety formula   C20 H4 F24 Na4 O8             ?
Sum formula      C20 H4 F24 Na4 O8             C20 H4 F24 Na4 O8
Mr               920.19                        920.19
Dx,g cm-3        2.065                         2.064
Z                8                             8
Mu (mm-1)        2.803                         2.803
F000             3584.0                        3584.0
F000'            3607.83
h,k,lmax         29,29,11                      29,29,11
Nref             5443[ 2895]                   5247
Tmin,Tmax        0.501,0.604                   0.224,0.753
Tmin'            0.434

Correction method= # Reported T Limits: Tmin=0.224 Tmax=0.753
AbsCorr = MULTI-SCAN

Data completeness= 1.81/0.96       Theta(max)= 68.403

                                                      wR2(reflections)=
R(reflections)= 0.0821( 4096)                         0.2541( 5247)

S = 0.993                    Npar= 505
```

The following ALERTS were generated. Each ALERT has the format
test-name_ALERT_alert-type_alert-level.
Click on the hyperlinks for more details of the test.

Alert level B

```
PLAT340_ALERT_3_B Low Bond Precision on  C-C Bonds ..............    0.01444 Ang.
```

Author Response: Several crystals of 1 have been tested. Unfortunately their quality is not excellent. The data in the present cif have been obtained analysing the best crystal we were able to find.

Alert level C

```
STRVA01_ALERT_4_C          Flack test results are ambiguous.
           From the CIF: _refine_ls_abs_structure_Flack    0.430
           From the CIF: _refine_ls_abs_structure_Flack_su    0.120
PLAT089_ALERT_3_C Poor Data / Parameter Ratio (Zmax < 18) ........       5.71 Note
PLAT213_ALERT_2_C Atom F5C             has ADP max/min Ratio .....        3.3 prolat
PLAT213_ALERT_2_C Atom C15             has ADP max/min Ratio .....        3.5 oblate
PLAT220_ALERT_2_C NonSolvent   Resd 1  F   Ueq(max)/Ueq(min) Range        3.5 Ratio
PLAT250_ALERT_2_C Large U3/U1 Ratio for Average U(i,j) Tensor ....        2.2 Note
PLAT911_ALERT_3_C Missing FCF Refl Between Thmin & STh/L=    0.600          9 Report
PLAT976_ALERT_2_C Check Calcd Resid. Dens.  0.80Ang From O8      .      -0.56 eA-3
PLAT976_ALERT_2_C Check Calcd Resid. Dens.  0.86Ang From O4      .      -0.51 eA-3
```

Alert level G

```
PLAT004_ALERT_5_G Polymeric Structure Found with Maximum Dimension          1 Info
PLAT242_ALERT_2_G Low    'MainMol' Ueq as Compared to Neighbors of         C4 Check
PLAT242_ALERT_2_G Low    'MainMol' Ueq as Compared to Neighbors of         C5 Check
PLAT242_ALERT_2_G Low    'MainMol' Ueq as Compared to Neighbors of         C9 Check
PLAT242_ALERT_2_G Low    'MainMol' Ueq as Compared to Neighbors of        C10 Check
PLAT242_ALERT_2_G Low    'MainMol' Ueq as Compared to Neighbors of        C14 Check
PLAT242_ALERT_2_G Low    'MainMol' Ueq as Compared to Neighbors of        C15 Check
PLAT242_ALERT_2_G Low    'MainMol' Ueq as Compared to Neighbors of        C20 Check
PLAT434_ALERT_2_G Short Inter HL..HL Contact F4A      ..F20A     .       2.79 Ang.
                                               y,1-x,1-z  =      3_566 Check
PLAT434_ALERT_2_G Short Inter HL..HL Contact F10B     ..F14A     .       2.81 Ang.
                                      -1/2+y,1/2-x,3/2-z  =      7_456 Check
PLAT434_ALERT_2_G Short Inter HL..HL Contact F15B     ..F20A     .       2.78 Ang.
                                                y,1-x,-z  =      3_565 Check
PLAT764_ALERT_4_G Overcomplete CIF Bond List Detected (Rep/Expd) .       1.18 Ratio
PLAT883_ALERT_1_G No Info/Value for _atom_sites_solution_primary .     Please Do !
PLAT912_ALERT_4_G Missing # of FCF Reflections Above STh/L=  0.600          1 Note
PLAT913_ALERT_3_G Missing # of Very Strong Reflections in FCF ....          1 Note
PLAT965_ALERT_2_G The SHELXL WEIGHT Optimisation has not Converged     Please Check
PLAT978_ALERT_2_G Number C-C Bonds with Positive Residual Density.          0 Info
```

0 **ALERT level A** = Most likely a serious problem - resolve or explain
1 **ALERT level B** = A potentially serious problem, consider carefully
9 **ALERT level C** = Check. Ensure it is not caused by an omission or oversight

```
 17 ALERT level G = General information/check it is not something unexpected

  1 ALERT type 1 CIF construction/syntax error, inconsistent or missing data
 18 ALERT type 2 Indicator that the structure model may be wrong or deficient
  4 ALERT type 3 Indicator that the structure quality may be low
  3 ALERT type 4 Improvement, methodology, query or suggestion
  1 ALERT type 5 Informative message, check
```

It is advisable to attempt to resolve as many as possible of the alerts in all categories. Often the minor alerts point to easily fixed oversights, errors and omissions in your CIF or refinement strategy, so attention to these fine details can be worthwhile. In order to resolve some of the more serious problems it may be necessary to carry out additional measurements or structure refinements. However, the purpose of your study may justify the reported deviations and the more serious of these should normally be commented upon in the discussion or experimental section of a paper or in the "special_details" fields of the CIF. checkCIF was carefully designed to identify outliers and unusual parameters, but every test has its limitations and alerts that are not important in a particular case may appear. Conversely, the absence of alerts does not guarantee there are no aspects of the results needing attention. It is up to the individual to critically assess their own results and, if necessary, seek expert advice.

Publication of your CIF in IUCr journals

A basic structural check has been run on your CIF. These basic checks will be run on all CIFs submitted for publication in IUCr journals (*Acta Crystallographica*, *Journal of Applied Crystallography*, *Journal of Synchrotron Radiation*); however, if you intend to submit to *Acta Crystallographica Section C* or *E* or *IUCrData*, you should make sure that full publication checks are run on the final version of your CIF prior to submission.

Publication of your CIF in other journals

Please refer to the *Notes for Authors* of the relevant journal for any special instructions relating to CIF submission.

PLATON version of 18/05/2022; check.def file version of 17/05/2022

Datablock 1 - ellipsoid plot

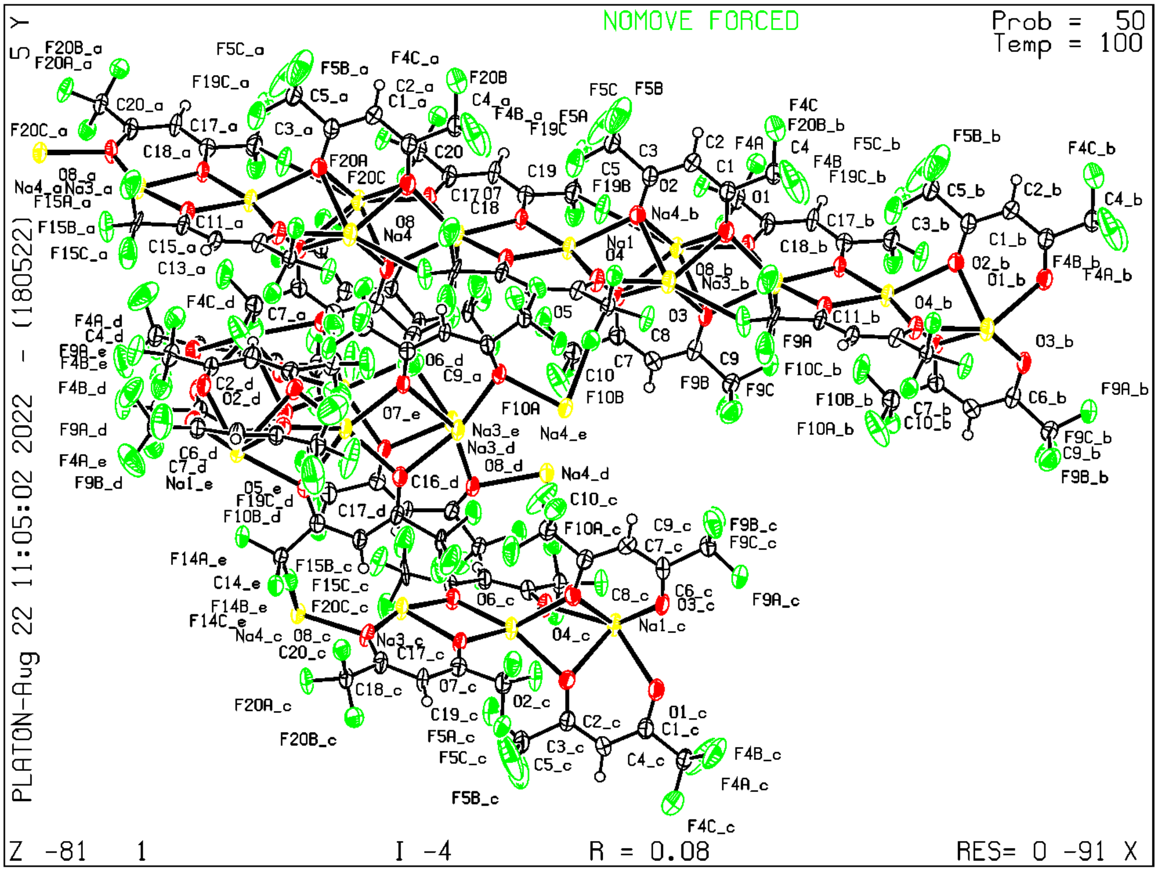

Supplement: Supplementary file 1 [file checkcif_1.pdf › Supporting/checkcif_1.pdf]
