# Supplementary material for: Sodium β-Diketonate Glyme Adducts as Precursors for Fluoride Phases: Synthesis, Characterization and Functional Validation
Source: Molecules. 2022 Sep 23;27(19):6282. doi: 10.3390/molecules27196282 (PMC9571527; doi:10.3390/molecules27196282)

checkCIF/PLATON report

Structure factors have been supplied for datablock(s) 2

THIS REPORT IS FOR GUIDANCE ONLY. IF USED AS PART OF A REVIEW PROCEDURE FOR PUBLICATION, IT SHOULD NOT REPLACE THE EXPERTISE OF AN EXPERIENCED CRYSTALLOGRAPHIC REFEREE.

No syntax errors found. CIF dictionary Interpreting this report

Datablock: 2

Bond precision: C-C = 0.0104 A Wavelength=1.54178

Cell:	a=11.4597(10)	b=17.0190(13)	c=21.8159(18)
	alpha=83.860(4)	beta=87.651(5)	gamma=71.840(4)
Temperature:	100 K		

	Calculated	Reported
Volume	4019.6(6)	4019.6(6)
Space group	P -1	P -1
Hall group	-P 1	-P 1
Moiety formula	C30 H6 F36 Na4 O12, 2(C12 H28 Na O6)	?
Sum formula	C54 H62 F36 Na6 O24	C54 H62 F36 Na6 O24
Mr	1916.98	1916.97
Dx,g cm-3	1.584	1.584
Z	2	2
Mu (mm-1)	1.839	1.839
F000	1936.0	1936.0
F000'	1946.93	
h,k,lmax	13,20,26	13,20,26
Nref	14851	14654
Tmin,Tmax	0.663,0.705	0.390,0.753
Tmin'	0.601	

Correction method= # Reported T Limits: Tmin=0.390 Tmax=0.753
AbsCorr = ?

Data completeness= 0.987 Theta(max)= 68.696

R(reflections)= 0.1584(10926) wR2(reflections)= 0.4627(14654)

S = 1.927 Npar= 1082

The following ALERTS were generated. Each ALERT has the format
test-name_ALERT_alert-type_alert-level.
Click on the hyperlinks for more details of the test.

Alert level A

PLAT084_ALERT_3_A High wR2 Value (i.e. > 0.25) .... 0.46 Report

Author Response: several crystals of 2 were tested and all resulted to be twins. The results reported in the article are those obtained by the best crystal we were able to find.

Alert level B

PLAT082_ALERT_2_B High R1 Value .... 0.16 Report

Author Response: several crystals of 2 were tested and all resulted to be twins. The results reported in the article are those obtained by the best crystal we were able to find.

PLAT097_ALERT_2_B Large Reported Max. (Positive) Residual Density 1.20 eA-3

Author Response: this density peak is due to disorded around one CF3 moiety

PLAT340_ALERT_3_B Low Bond Precision on C-C Bonds .... 0.01041 Ang.

Author Response: The observed low bond precision may be attributed to the not excellent quality of the data (twin crystals)

Alert level C

```
DIFMN02_ALERT_2_C  The minimum difference density is < -0.1*ZMAX*0.75
            _refine_diff_density_min given =     -0.892
            Test value =     -0.825
DIFMN03_ALERT_1_C  The minimum difference density is < -0.1*ZMAX*0.75
            The relevant atom site should be identified.
DIFMX02_ALERT_1_C  The maximum difference density is > 0.1*ZMAX*0.75
            The relevant atom site should be identified.
SHFSU01_ALERT_2_C  The absolute value of parameter shift to su ratio > 0.05
            Absolute value of the parameter shift to su ratio given   0.075
            Additional refinement cycles may be required.
PLAT080_ALERT_2_C Maximum Shift/Error ...........................       0.08 Why ?
PLAT098_ALERT_2_C Large Reported Min.  (Negative) Residual Density      -0.89 eA-3
PLAT213_ALERT_2_C Atom F241            has ADP max/min Ratio .....        3.4 prolat
PLAT220_ALERT_2_C NonSolvent   Resd 1  F   Ueq(max)/Ueq(min) Range        3.5 Ratio
PLAT234_ALERT_4_C Large Hirshfeld Difference F241      --C24      .       0.16 Ang.
PLAT906_ALERT_3_C Large K Value in the Analysis of Variance ......     21.121 Check
```

```
PLAT906_ALERT_3_C Large K Value in the Analysis of Variance ......      4.545 Check
PLAT906_ALERT_3_C Large K Value in the Analysis of Variance ......      2.185 Check
PLAT911_ALERT_3_C Missing FCF Refl Between Thmin & STh/L=    0.600         92 Report
```

Alert level G

```
PLAT180_ALERT_4_G Check Cell Rounding: # of Values Ending with 0 =          3 Note
PLAT242_ALERT_2_G Low    'MainMol' Ueq as Compared to Neighbors of         C4 Check
PLAT242_ALERT_2_G Low    'MainMol' Ueq as Compared to Neighbors of         C5 Check
PLAT242_ALERT_2_G Low    'MainMol' Ueq as Compared to Neighbors of         C9 Check
PLAT242_ALERT_2_G Low    'MainMol' Ueq as Compared to Neighbors of        C10 Check
PLAT242_ALERT_2_G Low    'MainMol' Ueq as Compared to Neighbors of        C14 Check
PLAT242_ALERT_2_G Low    'MainMol' Ueq as Compared to Neighbors of        C15 Check
PLAT242_ALERT_2_G Low    'MainMol' Ueq as Compared to Neighbors of        C19 Check
PLAT242_ALERT_2_G Low    'MainMol' Ueq as Compared to Neighbors of        C20 Check
PLAT242_ALERT_2_G Low    'MainMol' Ueq as Compared to Neighbors of        C24 Check
PLAT242_ALERT_2_G Low    'MainMol' Ueq as Compared to Neighbors of        C25 Check
PLAT242_ALERT_2_G Low    'MainMol' Ueq as Compared to Neighbors of        C29 Check
PLAT242_ALERT_2_G Low    'MainMol' Ueq as Compared to Neighbors of        C30 Check
PLAT434_ALERT_2_G Short Inter HL..HL Contact F252     ..F303     .       2.77 Ang.
                                                   1+x,y,z  =      1_655 Check
PLAT870_ALERT_4_G ALERTS Related to Twinning Effects Suppressed ..          ! Info
PLAT883_ALERT_1_G No Info/Value for _atom_sites_solution_primary .     Please Do !
PLAT912_ALERT_4_G Missing # of FCF Reflections Above STh/L=  0.600        106 Note
PLAT913_ALERT_3_G Missing # of Very Strong Reflections in FCF ....          1 Note
```

---

1 **ALERT level A** = Most likely a serious problem - resolve or explain
3 **ALERT level B** = A potentially serious problem, consider carefully
13 **ALERT level C** = Check. Ensure it is not caused by an omission or oversight
18 **ALERT level G** = General information/check it is not something unexpected

3 ALERT type 1 CIF construction/syntax error, inconsistent or missing data
21 ALERT type 2 Indicator that the structure model may be wrong or deficient
7 ALERT type 3 Indicator that the structure quality may be low
4 ALERT type 4 Improvement, methodology, query or suggestion
0 ALERT type 5 Informative message, check

It is advisable to attempt to resolve as many as possible of the alerts in all categories. Often the minor alerts point to easily fixed oversights, errors and omissions in your CIF or refinement strategy, so attention to these fine details can be worthwhile. In order to resolve some of the more serious problems it may be necessary to carry out additional measurements or structure refinements. However, the purpose of your study may justify the reported deviations and the more serious of these should normally be commented upon in the discussion or experimental section of a paper or in the "special_details" fields of the CIF. checkCIF was carefully designed to identify outliers and unusual parameters, but every test has its limitations and alerts that are not important in a particular case may appear. Conversely, the absence of alerts does not guarantee there are no aspects of the results needing attention. It is up to the individual to critically assess their own results and, if necessary, seek expert advice.

Publication of your CIF in IUCr journals

A basic structural check has been run on your CIF. These basic checks will be run on all CIFs submitted for publication in IUCr journals (*Acta Crystallographica*, *Journal of Applied Crystallography*, *Journal of Synchrotron Radiation*); however, if you intend to submit to *Acta Crystallographica Section C* or *E* or *IUCrData*, you should make sure that full publication checks are run on the final version of your CIF prior to submission.

Publication of your CIF in other journals

Please refer to the *Notes for Authors* of the relevant journal for any special instructions relating to CIF submission.

PLATON version of 18/05/2022; check.def file version of 17/05/2022

Datablock 2 - ellipsoid plot

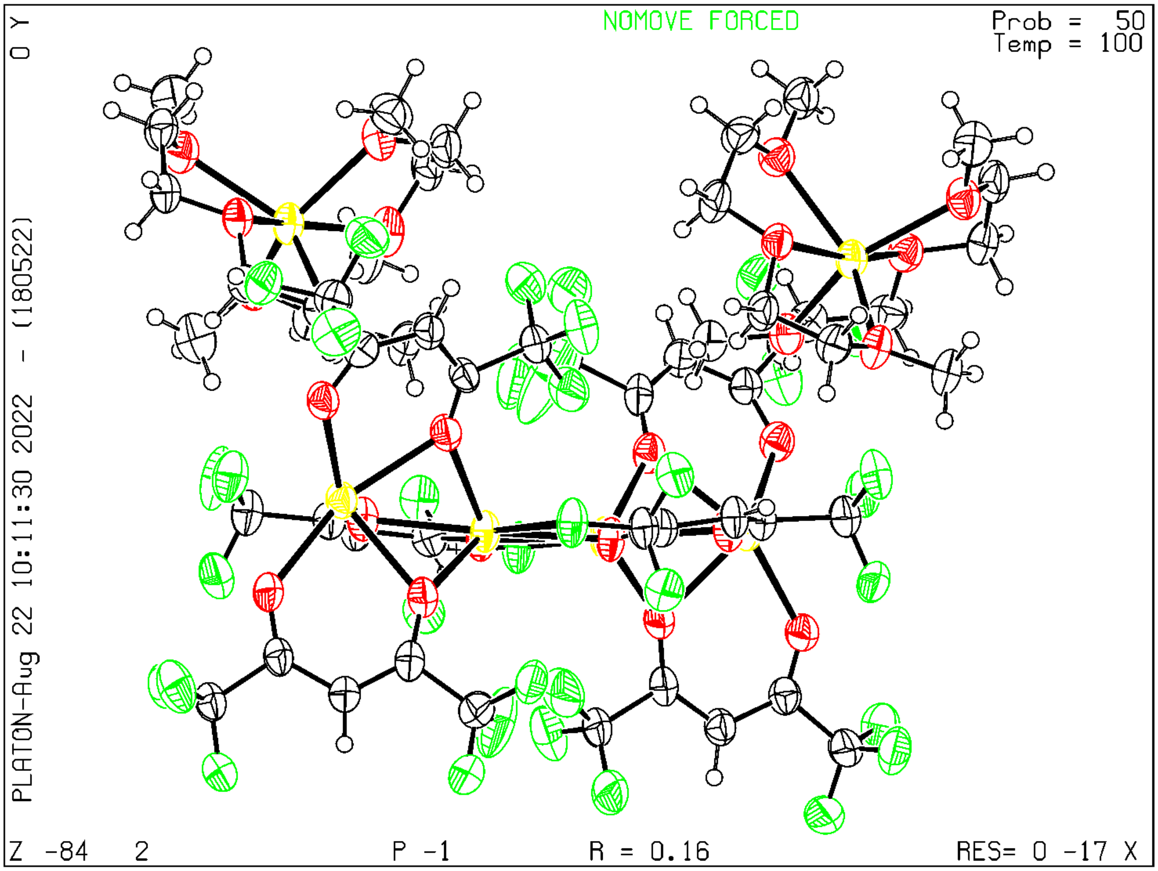

Supplement: Supplementary file 1 [file checkcif_2.pdf › Supporting/checkcif_2.pdf]
